# An MOF-Based Single-Molecule Propylene Nanotrap for Benchmark Propylene Capture from Ethylene

**DOI:** 10.1021/cbe.4c00102

**Published:** 2024-07-29

**Authors:** Jia-Xin Wang, Teng-Fei Zhang, Jiyan Pei, Di Liu, Yu-Bo Wang, Xiao-Wen Gu, Guodong Qian, Bin Li

**Affiliations:** State Key Laboratory of Silicon and Advanced Semiconductor Materials, School of Materials Science and Engineering, Zhejiang University, Hangzhou 310027, China

**Keywords:** porous materials, gas separation, nanotrap, propylene capture, ethylene separation

## Abstract

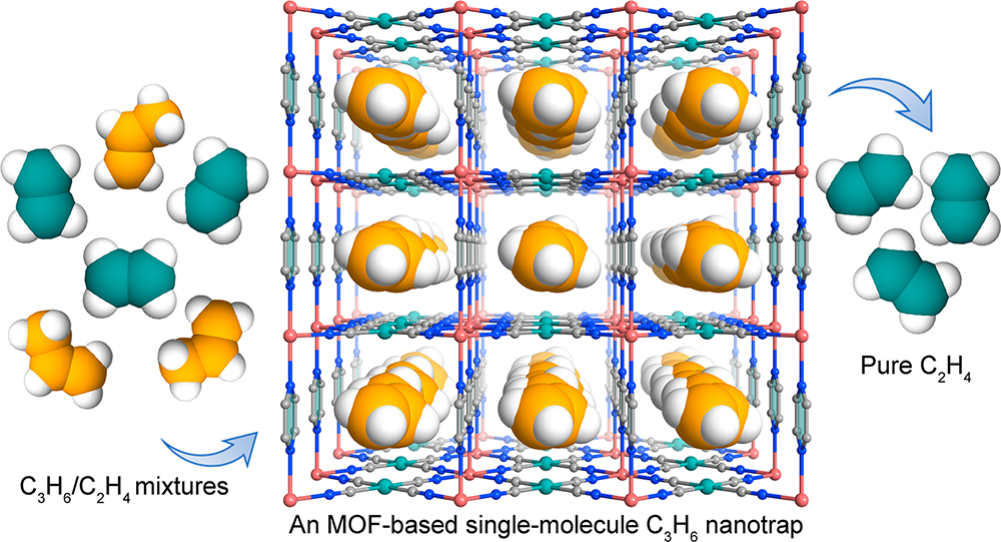

Highly selective capture and separation of propylene (C_3_H_6_) from ethylene (C_2_H_4_) presents one of the most crucial processes to obtain pure C_2_H_4_ in the petrochemical industry. The separation performance of current physisorbents is commonly limited by insufficient C_3_H_6_ binding affinity, resulting in poor low-pressure C_3_H_6_ uptakes or inadequate C_3_H_6_/C_2_H_4_ selectivities. Herein, we realize a unique single-molecule C_3_H_6_ nanotrap in an ultramicroporous MOF material (Co(pyz)[Pd(CN)_4_], ZJU-74a-Pd), exhibiting the benchmark C_3_H_6_ capture capacity at low-pressure regions. This MOF-based nanotrap features the sandwichlike strong multipoint binding sites and the perfect size match with C_3_H_6_ molecules, providing an ultrastrong C_3_H_6_ binding affinity with the maximal *Q*_st_ value (55.8 kJ mol^–1^). This affords the nanotrap to exhibit one of the highest C_3_H_6_ uptakes at low pressures (60.5 and 103.8 cm^3^ cm^–3^ at 0.01 and 0.1 bar) and record-high C_3_H_6_/C_2_H_4_ selectivity (23.4). Theoretical calculations reveal that the perfectly size-matched pore cavities combined with sandwichlike multibinding sites enable this single-molecule C_3_H_6_ nanotrap to maximize the C_3_H_6_ binding affinity, mainly accounting for its record low-pressure C_3_H_6_ capture capacity and selectivity. Breakthrough experiments further confirm its excellent separation capacity for actual 1/99 and 50/50 C_3_H_6_/C_2_H_4_ mixtures, affording the remarkably high pure C_2_H_4_ productivities of 17.1 and 3.4 mol kg^–1^, respectively.

## Introduction

Ethylene (C_2_H_4_) is an important raw material for the manufacture of many high-valued commodities including polyethylene, polyvinyl chloride, etc.^1−8^ It is estimated that the total global production of C_2_H_4_ exceeded 200 million tons in 2023.^9,10^ Industrially, C_2_H_4_ is primarily produced by steam cracking of the naphtha/ethane or methanol-to-olefins (MTO) process. However, these processes cannot produce pure C_2_H_4_ gas directly, in which C_2_H_4_ and propylene (C_3_H_6_) commonly coexist in the outlet gases. For example, the steam cracking of naphtha/ethane usually yields C_2_H_4_ gas as the main product, but inevitably contains a small amount of C_3_H_6_ (ranging from 1 to 20%).^11,12^ The MTO reactions can simultaneously produce C_3_H_6_ and C_2_H_4_ with a molar ratio of 1.0–2.5.^13,14^ Therefore, the selective capture and removal of C_3_H_6_ from C_2_H_4_ (especially trace amounts of C_3_H_6_) presents a critical process to obtain high-purity C_2_H_4_ (≥99.95%) for downstream applications. At present, this separation is primarily achieved by extractive distillation and catalytic hydrogenation, which are energy-intensive and environmentally unfriendly.^15^ In this context, adsorptive separation by porous materials has been considered as one of the most promising technologies to replace traditional methods because of its low energy footprints and high efficiency.^16−21^

Microporous metal–organic frameworks (MOFs) have attracted intensive attention for gas separations due to their predictable structures, tunable pore sizes/shapes, and easily accessible functionalities.^22−30^ Over the past two decades, a large number of MOF materials have been demonstrated to show impressive separation performances for a broad range of gas mixtures.^31−39^ Some MOFs have also been developed for efficient C_3_H_6_/C_2_H_4_ separation.^40−43^ However, most of them still suffer from either low gas uptake or poor selectivity, especially when C_3_H_6_ gas is present at trace levels. This is because both C_3_H_6_ and C_2_H_4_ molecules have the same unsaturated C=C bonds and similar molecular sizes (Table S2), making them very difficult to be distinguished for porous materials. Some MOFs have recently been designed by the strategies of fine-tuning pore sizes or functionalities to improve the C_3_H_6_/C_2_H_4_ separation performance.^44−49^ For example, great efforts have been dedicated to incorporating polar functional sites or open metal sites (OMSs) into MOFs to strengthen the C_3_H_6_ adsorption affinity and enhance C_3_H_6_/C_2_H_4_ selectivity. The well-known materials are those MOFs with high densities of OMSs such as MIL-101(Cr), Mn-dtzip, iso-MOF-4, and Ni-MOF-74.^42−45^ While these OMSs can form strong π-complexation interactions with unsaturated C=C bonds to target large low-pressure C_3_H_6_ uptake capacities, they are unable to effectively recognize these two gases with high selectivity because of their large pores and the same C=C bonds in both C_3_H_6_ and C_2_H_4_ molecules. In addition, their large pores with isolated OMSs also limit their C_3_H_6_ binding affinities to be moderate (30–40 kJ mol^–1^) in most cases.^43−46^ These limitations greatly restrict their C_3_H_6_/C_2_H_4_ selectivities lower than 10. On the other hand, the fine-tuning of pore sizes in some reported MOFs provides an efficient size differentiation to target high separation performance.^47−49^ However, their relatively weak C_3_H_6_ binding affinities (∼30 kJ mol^–1^) and low pore volumes commonly result in low C_3_H_6_ uptake capacities at low-pressure regions, greatly delimiting their capture capacities for trace amounts of C_3_H_6_. Apparently, simple pore size tuning or pore functionalization with OMSs in MOFs cannot achieve an ultrastrong C_3_H_6_ binding affinity, resulting in inadequate low-pressure C_3_H_6_ uptakes or poor selectivities. It is still very challenging to develop ideal porous materials with ultrastrong C_3_H_6_ binding sites for targeting ultrahigh low-pressure C_3_H_6_ capture capacity and selectivity.

**Figure 1:**
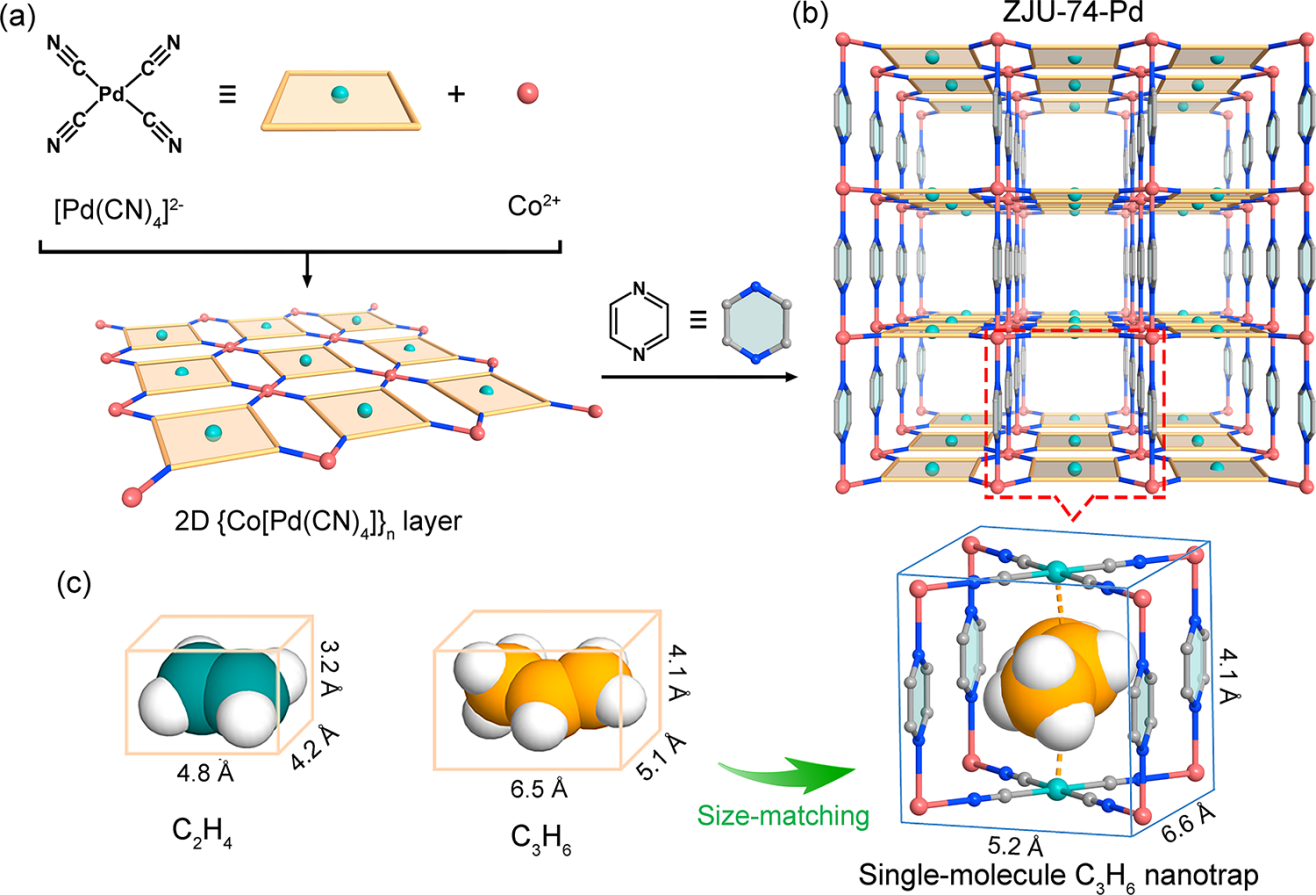
(a and b) Single-crystal structure of ZJU-74-Pd, showing the 1D square-grid pore channels. (c) Illustration of the molecular sizes of C_3_H_6_ (4.1 × 5.1 × 6.5 Å^3^) and C_2_H_4_ (3.2 × 4.2 × 4.8 Å^3^) as well as the single-molecule nanotrap (4.1 × 5.2 × 6.6 Å^3^), revealing the perfect size matching between C_3_H_6_ molecule and the nanotrap. Color code: Co (pink), Pd (yellow), N (blue), C (gray), and H (white).

To overcome the above challenge, it is highly important to design and realize ideal ultrastrong C_3_H_6_ binding sites for maximizing the low-pressure C_3_H_6_ capture capacity and selectivity. Recent studies have shown that the design of suitable nanotraps with well-matched sizes and multiple strong binding sites can significantly improve targeted gas binding affinity, thus resulting in ultrahigh low-pressure gas uptakes and selectivities.^50−52^ This strategy has been well realized in some MOF-based nanotraps for high C_2_H_2_, C_3_H_4_, Xe, and CH_4_ low-pressure adsorption and their related gas separations.^53−55^ Inspired by this progress, we speculated that if we can construct a suitable nanotrap within porous MOFs to realize ultrastrong C_3_H_6_ binding affinity, it is of high potential to afford the desired ultrahigh low-pressure C_3_H_6_ capture capacity and selectivity for C_3_H_6_/C_2_H_4_ separation. Herein, we target this matter in an MOF-based single-molecule C_3_H_6_ nanotrap, Co(pyz)[Pd(CN)_4_] (ZJU-74a-Pd, pyz = pyrazine).^55^ This material features a sandwichlike binding nanotrap, which is constructed by two oppositely adjacent OMSs and four pyrazine ligands (Figure 1). This nanotrap exhibits a perfect cavity size (4.1 × 5.2 × 6.6 Å^3^) to match much better with the C_3_H_6_ molecule (4.1 × 5.1 × 6.5 Å^3^) than C_2_H_4_.^56^ Combined with its sandwichlike binding model, this nanotrap thus enables the two oppositely adjacent OMSs and multiple N sites to provide dual and strong interactions with the C_3_H_6_ molecule, affording unprecedented and ultrastrong C_3_H_6_ binding affinity with one of the highest *Q*_st_ values (55.8 kJ mol^–1^) for C_3_H_6_ capture and separation, as is also supported by theoretical simulations of the gas binding. As a result, ZJU-74a-Pd exhibits one of the highest C_3_H_6_ uptake capacities at low-pressure regions (60.5 and 103.8 cm^3^ cm^–3^ at 0.01 and 0.1 bar) and very high C_3_H_6_/C_2_H_4_ selectivity (23.4) at ambient conditions, exceeding all the best-performing MOFs reported so far. The extraordinary separation performance of ZJU-74a-Pd was confirmed by actual breakthrough experiments for 1/99 and 50/50 (v/v) C_3_H_6_/C_2_H_4_ mixtures, providing the remarkably high C_2_H_4_ productivities of 17.1 and 3.4 mol kg^–1^, respectively.

## Experimental Section

### Synthesis of ZJU-74-Pd

The crystalline samples were prepared according to the previously reported literature.^55^ An aqueous solution (2 mL) of K_2_[Pd(CN)_4_]·3H_2_O (0.5 mmol) was added to a methanol/water solution (1/1, 10 mL) of Co(NO_3_)_2_·6H_2_O (0.5 mmol) and pyz (0.5 mmol) at 30 °C, and the resulting light pink precipitate was obtained under constant stirring for 3 h.

### Gas Sorption Measurements

Micromeritics ASAP 2020 and ASAP 2460 surface area analyzers were used to measure C_3_H_6_, C_2_H_4_, and N_2_ adsorption isotherms, respectively. To remove all the guest solvents in the framework, the fresh sample of ZJU-74-Pd was guest-exchanged with dry acetone at least 10 times within 2 days, filtered and degassed at room temperature (RT) for 12 h and then at 323 K for another 12 h until the outgassing rate was 5 μmHg min^–1^ before measurement. The N_2_ sorption measurement was maintained at 77 K with liquid nitrogen. A Julabo water bath was used to keep the adsorption tube at constant temperatures of 273, 296, and 313 K, respectively.

### Column Breakthrough Experiments

The breakthrough experiments for 1/99 and 50/50 (v/v) C_3_H_6_/C_2_H_4_ mixtures were performed in dynamic gas breakthrough equipment at 298 K and 1 bar. The activated ZJU-74a-Pd (0.3796 g) particles were prepared and packed into a stainless steel column (4.0 mm inner diameter × 65 mm). The gas flows were controlled at the inlet by using a mass flow meter at 1.5 mL min^–1^, and a gas chromatograph (thermal conductivity detector (TCD), detection limit 0.1 ppm) continuously monitored the effluent gas from the adsorption bed. The standard gases were used to calibrate the concentration of the outlet gas. Before every breakthrough experiment, the sample could be generated by a He flow (10 mL min^–1^) for 3 h at 323 K. Subsequently, the column was allowed to equilibrate at the measurement rate before we switched the gas flow.

## Results and Discussion

The crystalline ZJU-74-Pd sample was readily prepared according to our previously reported literature.^55^ Its high purity was confirmed by powder X-ray diffraction (PXRD) analysis (Figure S1). The crystal structure of ZJU-74-Pd has been already determined by single-crystal X-ray diffraction (SCXRD) analysis in our previous work. As depicted in Figure 1a,b, each Co(II) ion coordinates with four N atoms of [Pd(CN)_4_]^2–^ anion units to form a two-dimensional (2D) square-grid {Co[Pd(CN)_4_]}_*n*_ planar layer, which is further pillared by the pyrazine linkers to generate a 3D framework. Close inspection of this framework reveals that there exist numerous cagelike pore cavities along the pore channels in ZJU-74-Pd. Each pore cavity is constructed by two oppositely adjacent [Pd(CN)_4_]^2–^ units and four surrounding pyrazine ligands (Figure 1c). Most importantly, the pore cavity size is estimated to have a 3D size of 4.1 × 5.2 × 6.6 Å^3^ (after subtracting the van der Waals radius), which matches much better with the larger C_3_H_6_ molecule (4.1 × 5.1 × 6.5 Å^3^) than C_2_H_4_ (3.2 × 4.2 × 4.8 Å^3^).^56^ This makes each pore cavity serve as an ideal single-molecule trap for the capture of a single C_3_H_6_ molecule. Due to the better size match with the C_3_H_6_ molecule, two oppositely adjacent OMSs and eight N atoms from the adjacent [Pd(CN)_4_]^2–^ units in each nanotrap can create a unique sandwichlike multibinding model to optimize and maximize C_3_H_6_ binding energy. Further, such strong nanotraps are found to be densely and orderly distributed around pore channels along the *b* axis, potentially resulting in the dense packing of C_3_H_6_ to improve gas uptakes. Taken together, the perfectly size-matched pore cavities combined with sandwichlike multibinding sites within ZJU-74-Pd enable it to serve as a unique single-molecule C_3_H_6_ nanotrap, potentially providing the ultrastrong C_3_H_6_ binding affinity for targeting both high C_3_H_6_ capture capacity and selectivity at low pressures.

The permanent porosity of ZJU-74a-Pd was exclusively determined by nitrogen (N_2_) adsorption isotherms at 77 K and 1 bar with a N_2_ uptake of 158.4 cm^3^ g^–1^, affording a Brunauer–Emmett–Teller (BET) surface area of 590 m^2^ g^–1^ (Figure S2). The pore size distribution of ZJU-74a-Pd, determined by the Horvath–Kawazoe method, was calculated to be 3.8 Å (Figure S3). All these results are in good accordance with the previous literature,^55^ further confirming the high purity of the ZJU-74a-Pd sample.

**Figure 2:**
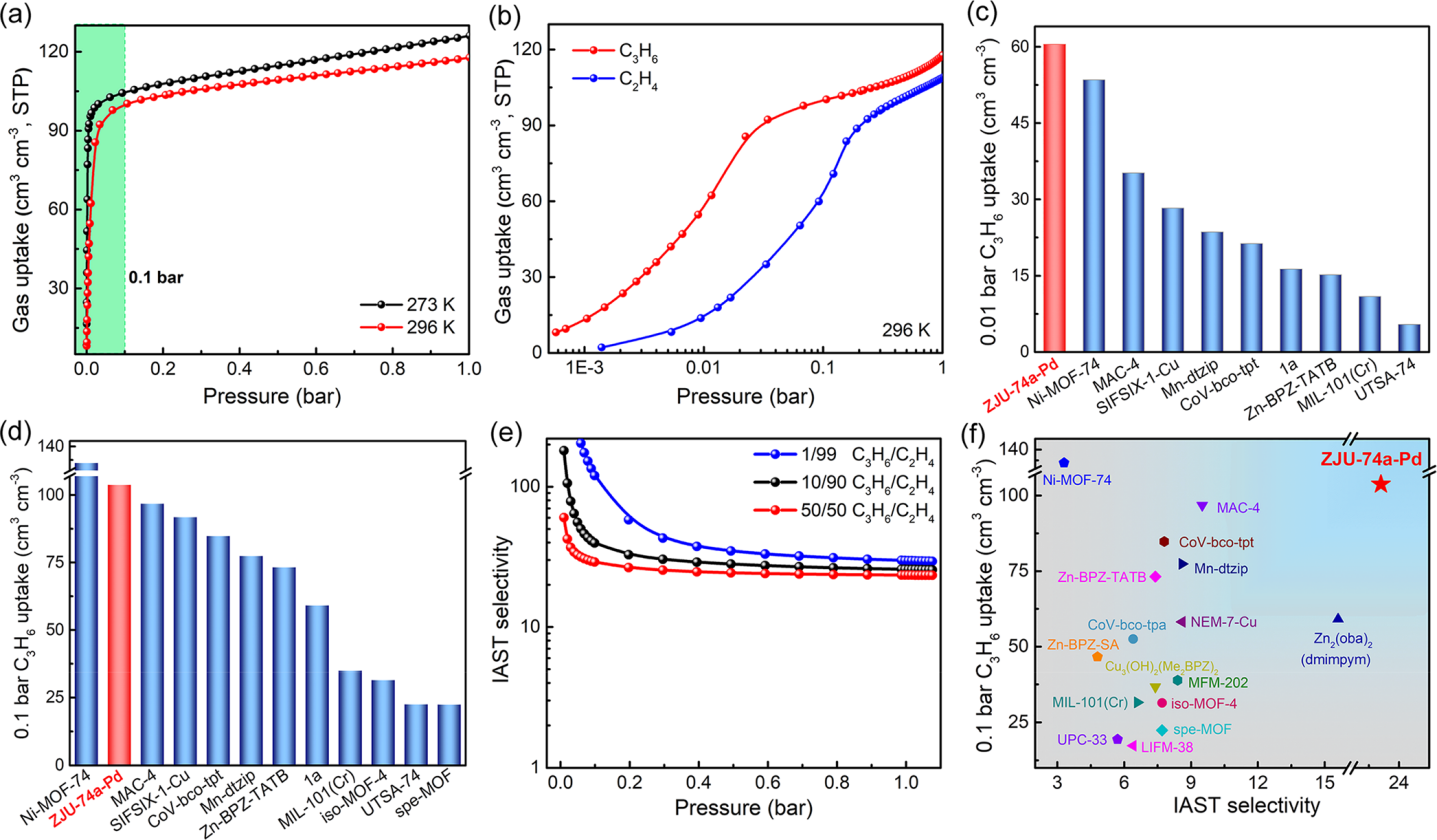
(a) Single-component gas adsorption isotherms of C_3_H_6_ for ZJU-74a-Pd at 273 and 296 K up to 1 bar. (b) Adsorption isotherms of C_3_H_6_ and C_2_H_4_ for ZJU-74a-Pd at 296 K up to 1 bar. (c and d) Comparison of C_3_H_6_ uptakes at 0.01 and 0.1 bar for ZJU-74a-Pd and other representative MOFs. (e) IAST selectivities of ZJU-74a-Pd for 1/99 (blue), 10/90 (black), and 50/50 (red) C_3_H_6_/C_2_H_4_ mixtures at 296 K up to 1 bar. (f) Comparison of C_3_H_6_ uptakes at 0.1 bar and 50/50 C_3_H_6_/C_2_H_4_ selectivities for ZJU-74a-Pd and other indicated MOFs.

Encouraged by the unique single-molecule C_3_H_6_ nanotrap observed in ZJU-74a-Pd, we first examined single-component gas adsorption isotherms for C_3_H_6_ and C_2_H_4_ at different temperatures up to 1 bar (Figure 2a), and the gas desorption isotherms are presented in Figures S4 and S5 accordingly. As shown in Figure 2a,b, ZJU-74a-Pd exhibits extremely steep C_3_H_6_ adsorption isotherms at both 273 and 296 K, reaching almost saturated uptakes below 0.1 bar. Particularly, ZJU-74a-Pd exhibits by far the highest C_3_H_6_ uptake of 60.5 cm^3^ cm^–3^ at 0.01 bar and 296 K (Figure 2c), surpassing all the reported MOFs such as Ni-MOF-74 (53.5 cm^3^ cm^–3^),^42^ MAC-4 (35.2 cm^3^ cm^–3^),^57^ SIFSIX-1-Cu (28.3 cm^3^ cm^–3^), and UTSA-74 (5.4 cm^3^ cm^–3^).^58^ The C_3_H_6_ uptake can further increase to 103.8 cm^3^ cm^–3^ at 296 K and 0.1 bar, which also exceeds all the top-performing MOFs except Ni-MOF-74 (Figure 2d and Table S3),^59,60^ such as MAC-4 (96.8 cm^3^ cm^–3^),^57^ SIFSIX-1-Cu (91.8 cm^3^ cm^–3^),^58^ Mn-dtzip (77.4 cm^3^ cm^–3^),^44^ MIL-101(Cr) (34.9 cm^3^ cm^–3^),^43^ and spe-MOF (22.4 cm^3^ cm^–3^).^46^ In contrast, the low-pressure C_2_H_4_ uptake of ZJU-74a-Pd is much lower than that of C_3_H_6_, with a much smaller uptake of 14.2 cm^3^ cm^–3^ at 0.01 bar. These results indicate that this well-matched nanotrap can selectively and reversibly take up much greater amounts of C_3_H_6_ over C_2_H_4_ at low-pressure regions. Such large adsorption discrepancies between C_3_H_6_ and C_2_H_4_ observed in ZJU-74a-Pd can be well supported by the experimental adsorption heat (*Q*_st_), where the *Q*_st_ value of C_3_H_6_ (55.8 kJ mol^–1^) at zero coverage is much higher than that of C_2_H_4_ (30.5 kJ mol^–1^) (Figure S6). It is worth noting that this C_3_H_6_*Q*_st_ value of ZJU-74a-Pd is the highest among all the MOF materials reported so far (Figure S7), even higher than that of some promising materials with strong OMSs such as Ni-MOF-74 (54.5 kJ mol^–1^),^42^ MIL-101(Cr) (34.3 kJ mol^–1^),^43^ iso-MOF-4 (30.9 kJ mol^–1^),^45^ and spe-MOF (29.6 kJ mol^–1^),^46^ which further confirms its ultrastrong C_3_H_6_ binding affinity. Cycling experiments were performed to investigate the regeneration ability of ZJU-74a-Pd for C_3_H_6_ adsorption. As shown in Figure S8, the almost unchanged C_3_H_6_ adsorption capacities for ZJU-74a-Pd indicate its good cycling ability for C_3_H_6_ adsorption.

Ideal adsorbed solution theory (IAST) was further performed to estimate the separation ability of ZJU-74a-Pd for different ratios of C_3_H_6_/C_2_H_4_ mixtures (Figures 2e and S9). Owing to its ultrahigh C_3_H_6_ capture uptakes and large C_3_H_6_/C_2_H_4_ adsorption differences at low pressures, ZJU-74a-Pd exhibits the exceptionally high C_3_H_6_/C_2_H_4_ selectivities of 302.8, 106.5, and 60.2 at low-pressure regions and 296 K for 1/99, 10/90, and 50/50 gas mixtures, respectively. With the gas pressure increased, the C_3_H_6_/C_2_H_4_ selectivity gradually decreases to 29.4, 25.5, and 23.4 at 1 bar (Figure 2e), respectively. We note that these C_3_H_6_/C_2_H_4_ selectivities of ZJU-74a-Pd at 1 bar are much higher than those of all the top-performing materials, including Zn_2_(oba)_2_(dmimpym) (15.6),^47^ MAC-4 (9.5),^57^ Mn-dtzip (8.6),^44^ iso-MOF-4 (7.7),^45^ spe-MOF (7.7),^46^ and Ni-MOF-74 (3.3).^44^ As we know, both high C_3_H_6_ uptake capacity and adsorption selectivity are two critical factors in determining the final separation performance for C_3_H_6_/C_2_H_4_ separation. Therefore, we comprehensively compared the C_3_H_6_ uptake and C_3_H_6_/C_2_H_4_ selectivity of ZJU-74a-Pd with the indicated best-performing MOFs. As illustrated in Figure 2f, ZJU-74a-Pd shows the best combination of simultaneously high C_3_H_6_ uptake and C_3_H_6_/C_2_H_4_ selectivity at 0.1 bar and 296 K, setting up a new benchmark for C_3_H_6_/C_2_H_4_ separation.

**Figure 3:**
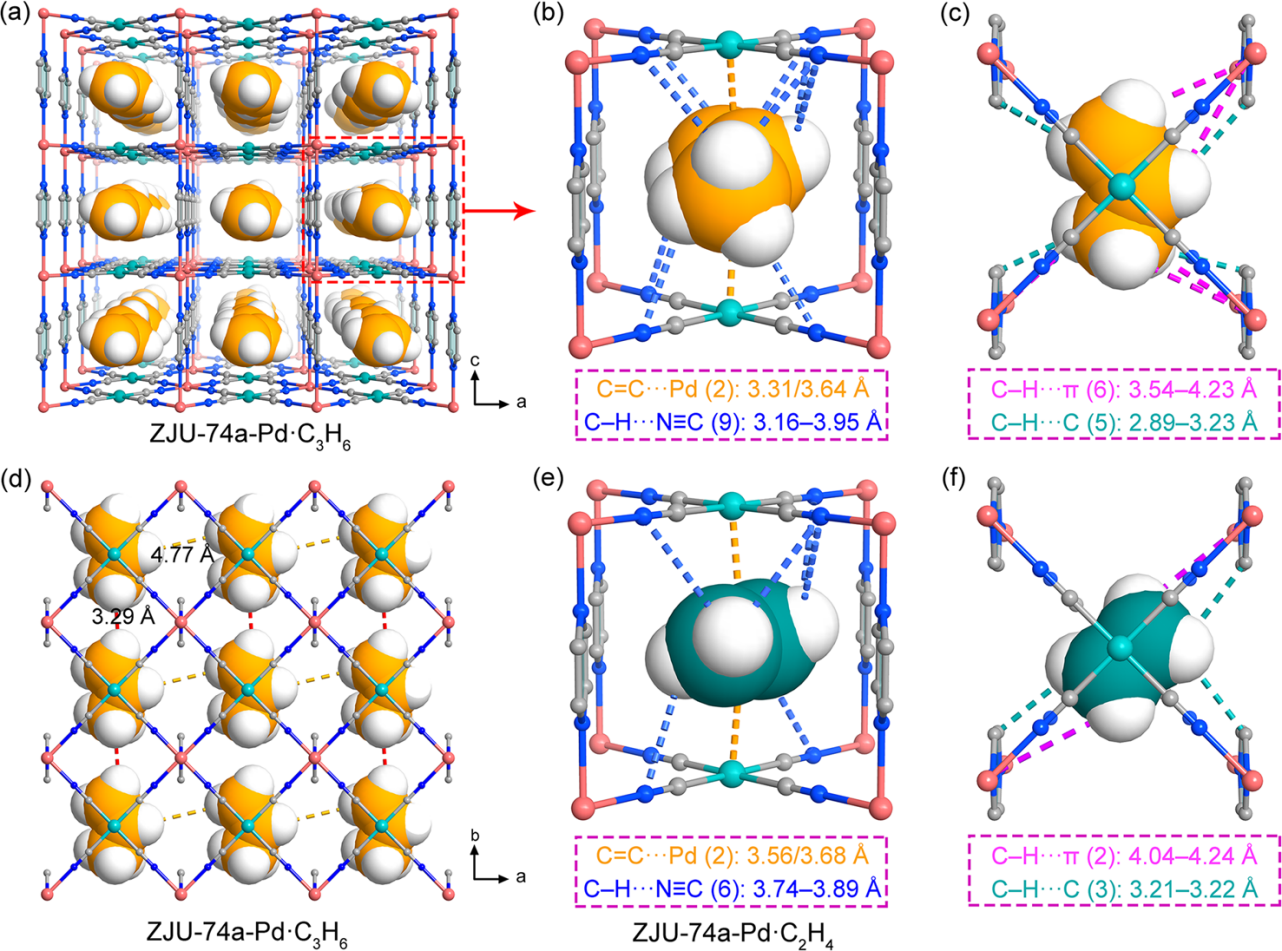
(a) Illustration of C_3_H_6_ adsorption sites in ZJU-74a-Pd, determined by GCMC calculations. (b and c) The preferential C_3_H_6_ adsorption site in ZJU-74a-Pd viewed along the *b* and *c* axes. (d) Dense packing of the adsorbed C_3_H_6_ molecules within ZJU-74a-Pd viewed along the *c* axis. (e and f) The preferential C_2_H_4_ adsorption site in ZJU-74a-Pd viewed along the *b* and *c* axes.

To gain a deep insight into the record C_3_H_6_ low-pressure uptakes and selectivities observed in ZJU-74a-Pd, we implemented grand canonical Monte Carlo (GCMC) simulations to determine the adsorption sites of C_3_H_6_ and C_2_H_4_ molecules within this nanotrap. As illustrated in Figures 3a and S12, both C_3_H_6_ and C_2_H_4_ are primarily located at the center of the nanotrap, which is formed by two oppositely adjacent OMSs and four surrounding pyrazine ligands. Each nanotrap can only adsorb one C_3_H_6_ molecule through dual π-complexation and multiple van der Waals (vdW) interactions, indicating a single-molecule C_3_H_6_ trapping behavior (Figure 3a). Within each single-molecule nanotrap, the adsorbed C_3_H_6_ molecule binds with two adjacent open Pd(II) sites in the short distances of 3.31 and 3.64 Å. This distance is even shorter than the sum of the vdW radii of the C_3_H_6_ (2.34 Å) molecule and Pd (1.69 Å) atom, indicating the strong binding affinity between the C_3_H_6_ molecule and OMSs. Moreover, the C_3_H_6_ molecule can also interact with seven surrounding N atoms from [Pd(CN)_4_]^2–^ anion units to form nine C–H···N≡C (H···N, 3.16–3.95 Å) interactions (Figure 3b). Due to the perfectly matched pore-cavity sizes, there also exist multiple C–H···π (H···π, 3.54–4.23 Å) and C–H···C (H···C, 2.89–3.23 Å) supramolecular interactions between the C_3_H_6_ molecule and four neighboring pyrazine linkers (Figures 3c and S13), further enhancing the C_3_H_6_ gas binding energy. In addition, owing to the dense distribution of these strong nanotraps within ZJU-74a-Pd, the adsorbed C_3_H_6_ molecules contact with each other in close proximity, with short distances of 3.29 Å along the *b* axis and 4.77 Å along the *a* axis (Figure 3d). This indicates a highly dense packing of C_3_H_6_ molecules within the framework of ZJU-74a-Pd, which also contributes to its ultrahigh low-pressure C_3_H_6_ capture capacity. Full occupancy of this strong single-molecule nanotrap corresponds to a gas uptake of 95.2 cm^3^ cm^–3^, which is very close to the experimental C_3_H_6_ uptake (103.8 cm^3^ cm^–3^) at 296 K and 0.1 bar. Taken together, the perfect size match and multiple strong binding sites within this single-molecule nanotrap can not only contribute to the ultrastrong C_3_H_6_ binding affinity but also result in a highly dense packing, thus maximizing the low-pressure C_3_H_6_ uptake capacities.

In comparison, the C_2_H_4_ molecule interacts more weakly with the framework because of its smaller molecular size than C_3_H_6_, making it mismatch with the nanotrap. As shown in Figure 3e, the C_2_H_4_ molecule binds with two adjacent open Pd(II) sites in a longer average Pd(II)···C=C distance (3.62 Å) and interacts with fewer N atoms and pyrazine rings through the smaller number of C–H···N≡C (H···N, 3.74–3.88 Å), C–H···π (H···π, 4.04 and 4.24 Å), and C–H···C (H···C, 3.21–3.22 Å) interactions (Figures 3f and S14). This size mismatch between the C_2_H_4_ molecule and the ZJU-74a-Pd nanotrap leads to the much smaller C_2_H_4_ binding affinity and thus much lower gas uptakes compared with that of C_3_H_6_, as is also verified by the much larger calculated binding energy of C_3_H_6_ (47.3 kJ mol^–1^) than that of C_2_H_4_ (34.5 kJ mol^–1^). All of these calculation results can explain the record low-pressure C_3_H_6_ uptake capacity and selectivity observed in ZJU-74a-Pd qualitatively.

**Figure 4:**
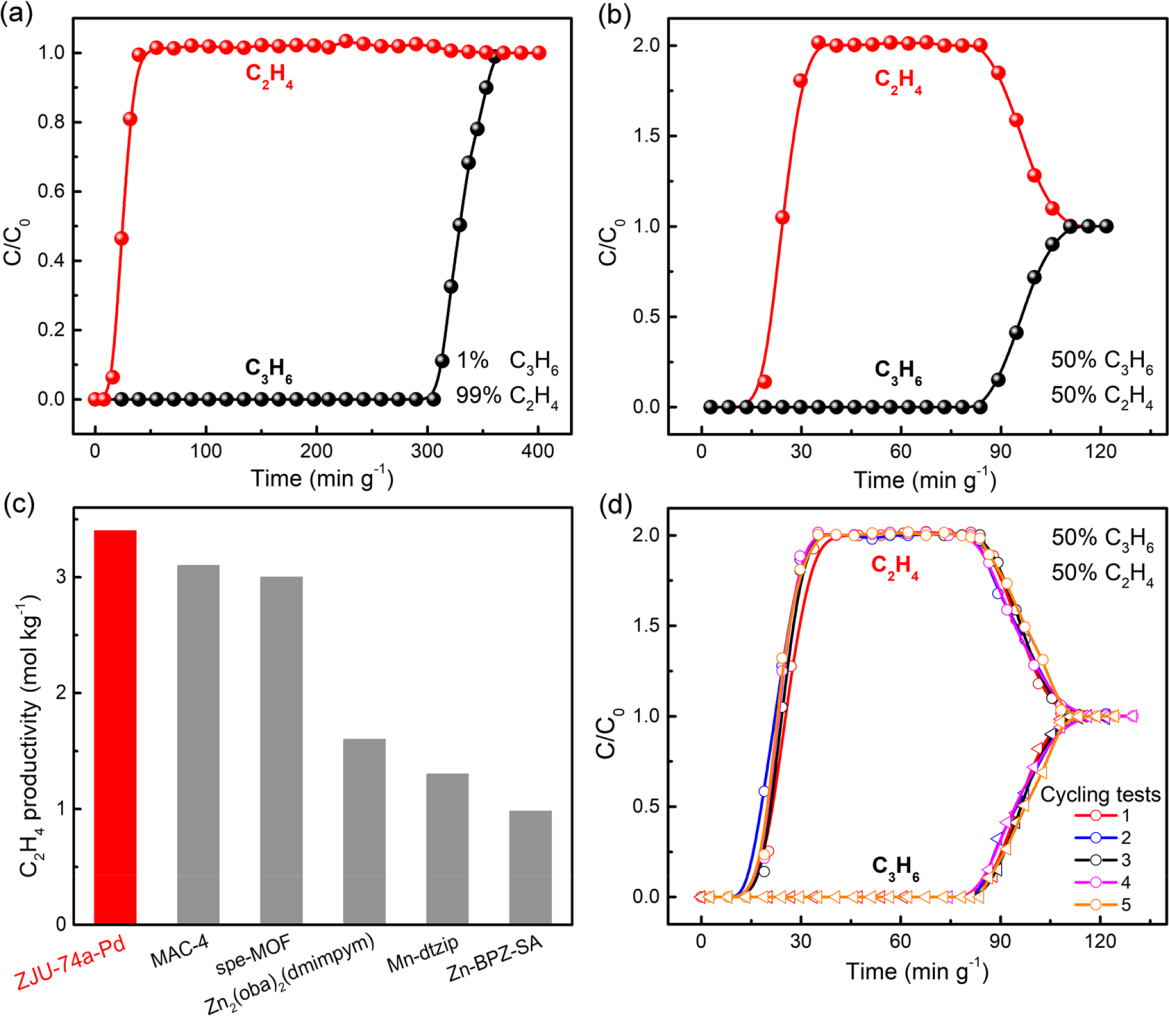
(a and b) Dynamic breakthrough experiments on ZJU-74a-Pd for 1/99 and 50/50 (v/v) C_3_H_6_/C_2_H_4_ mixtures with a flow rate of 1.5 mL min^–1^ under ambient conditions. (c) Comparison of pure C_2_H_4_ productivities (mol kg^–1^) for ZJU-74a-Pd and other top-performing MOFs. (d) Cycling breakthrough curves of ZJU-74a-Pd for a 50/50 C_3_H_6_/C_2_H_4_ mixture.

To validate the extraordinary separation performance of ZJU-74a-Pd for actual 1/99 and 50/50 C_3_H_6_/C_2_H_4_ mixtures, dynamic breakthrough experiments were performed on a packed column of activated ZJU-74a-Pd at ambient conditions. Owing to the record low-pressure uptake capacity and selectivity, ZJU-74a-Pd can efficiently capture and remove trace amounts of C_3_H_6_ from a 1/99 C_3_H_6_/C_2_H_4_ mixture to directly produce pure C_2_H_4_. During this breakthrough process, the C_2_H_4_ gas first eluted out from the absorber bed at 7.8 min g^–1^, producing pure C_2_H_4_ (>99.95%) containing undetectable C_3_H_6_ (the detection limit of the instrument is 0.01%). In contrast, C_3_H_6_ gas did not break out until 306.7 min g^–1^ due to its larger adsorption capacity. The high-purity C_2_H_4_ (>99.95%) obtained from this single breakthrough process was determined to be 17.1 mol kg^–1^ (Figures 4a and S15). Moreover, a highly efficient separation for a 50/50 gas mixture was also accomplished by ZJU-74a-Pd (Figure 4b), in which C_2_H_4_ first passed through the absorber bed at 13.6 min g^–1^ while C_3_H_6_ was retained in the packed column at 84.5 min g^–1^. Therefore, the high-purity C_2_H_4_ (>99.95%) obtained from a single process was calculated to be 3.4 mol kg^–1^ (Figure S16), which is higher than most of the best-performing MOFs reported, including MAC-4 (3.2 mol kg^–1^),^57^ spe-MOF (3.0 mol kg^–1^),^46^ Zn_2_(oba)_2_(dmimpym) (1.6 mol kg^–1^),^47^ and Mn-dtzip (1.2 mol kg^–1^)^44^ (Figure 4c). According to the breakthrough curves on a 50/50 gas mixture, the C_3_H_6_/C_2_H_4_ dynamic selectivity of ZJU-74a-Pd was assessed to be 8.7. Subsequently, cycling breakthrough experiments for C_3_H_6_/C_2_H_4_ mixtures were implemented under ambient conditions to evaluate the recyclable ability of ZJU-74a-Pd (Figure 4d). As shown in Figures S17 and S18, the separation performance of ZJU-74a-Pd can be preserved over five continuous cycles, verifying its excellent recyclability for this separation. These results have demonstrated that ZJU-74a-Pd can be recycled for repeated separation cycles without any loss of the framework integrity and separation performance. Further, the framework of ZJU-74-Pd also exhibits excellent chemical and thermal stabilities (Figure S19), which can retain its structural integrity in water and various pH solutions between 1 and 12, as clearly revealed in our previous work.^55^ Overall, ZJU-74a-Pd exhibits extraordinary capture and separation capacities for the separation of both trace and large amounts of C_3_H_6_ from C_3_H_6_/C_2_H_4_ mixtures, making it a promising adsorbent for the separation applications toward both steam cracking and MTO reaction products.

## Conclusions

In summary, we have successfully realized a perfect MOF-based single-molecule C_3_H_6_ nanotrap (ZJU-74a-Pd) for benchmark C_3_H_6_ capture capacity at low-pressure regions. This MOF-based nanotrap shows the perfect size match and unique sandwichlike multipoint binding sites toward C_3_H_6_ molecules, providing one of the strongest C_3_H_6_ binding affinities. This maximizes the C_3_H_6_ capture and separation performance to afford one of the highest C_3_H_6_ uptakes at low-pressure regions and the record C_3_H_6_/C_2_H_4_ selectivity reported so far, making it a new benchmark for C_3_H_6_/C_2_H_4_ separation. We attribute this ultrastrong C_3_H_6_ capture capacity to the unique sandwichlike nanotrap binding model and size-matched pore environments, in which the oppositely adjacent OMSs, Lewis N sites, and pyrazine rings can synergistically and strongly interact with the C_3_H_6_ molecule to maximize the C_3_H_6_ binding affinity, as indicated by theoretical calculations. Dynamic breakthrough experiments further confirmed its excellent separation performance, verifying that ZJU-74a-Pd can efficiently capture and separate both trace and large amounts of C_3_H_6_ from C_3_H_6_/C_2_H_4_ mixtures with high C_2_H_4_ productivities. This work not only reports a unique MOF-based single-molecule C_3_H_6_ nanotrap for benchmark C_3_H_6_ capture and separation, it also offers some guidance to develop new MOF-based single-molecule nanotraps to improve separation performances for other gas mixtures.

## Supporting Information Available

Details for experimental procedures, IAST calculations, powder X-ray diffraction patterns, GCMC simulations, gas equilibrium adsorption capacity, and heat of adsorption (*Q*_st_) calculations (PDF)
