# Supplementary material for: An MOF-Based Single-Molecule Propylene Nanotrap for Benchmark Propylene Capture from Ethylene
Source: Chem Bio Eng. 2024 Jul 29;1(11):952–9. doi: 10.1021/cbe.4c00102 (PMC11835284; doi:10.1021/cbe.4c00102)
Supplement: Supplementary file 1 — be4c00102_si_001.pdf [file be4c00102_si_001.pdf]

Supporting Information

A MOF-based Single-Molecule Propylene Nano-trap for Benchmark Propylene Capture from Ethylene

Jia-Xin Wang,‡ Teng-Fei Zhang,‡ Jiyan Pei,* Di Liu, Yu-Bo Wang, Xiao-Wen Gu, Guodong Qian, and Bin Li*

State Key Laboratory of Silicon and Advanced Semiconductor Materials, School of Materials Science and Engineering, Zhejiang University, Hangzhou 310027, China.

E-mail: bin.li@zju.edu.cn

1. General procedures and materials.

All starting reagents and solvents were purchased from commercial companies and used without further purification. Thermogravimetric analysis (TGA) was performed on a Netzsch TG209F3 instrument and the sample was heated under nitrogen atmosphere at a heating rate of 5 K min^{-1}. Powder X-ray diffraction (PXRD) patterns were collected in the 2θ = 2–45° range on an X'Pert PRO diffractometer with Cu K_{α} (λ = 1.542 Å) radiation at room temperature with a rate of 2° min^{-1}.

N_2 (99.99%), C_2H_4 (99.99%), C_3H_6 (99.99%), He (99.999%), and mixed gases of C_3H_6/C_2H_4 = 1/99, 50/50 (v/v) were purchased from Jin Gong Special Gas Company (China).

2. Single-crystal X-ray diffraction.

X-ray diffraction data of ZJU-74-Pd was collected on an Agilent Supernova CCD diffractometer equipped with graphite-monochromatic enhanced Mo-K_{α} radiation (λ = 0.71073 Å). The datasets were corrected by empirical absorption correction using spherical harmonics, implemented in the SCALE3 ABSPACK scaling algorithm. The structure was solved by direct methods and refined by full-matrix least-squares methods with the SHELX-97 program package.[1] The pyridine ligand is disordered. The crystal data have been presented in our previously reported literature.[2]

3. Fitting of pure component isotherms.

The pure component isotherm data for C_3H_6 and C_2H_4 in ZJU-74a-Pd at 296 K were fitted with the dual-Langmuir-Freundlich isotherm model

$$q = q_{A,sat}\frac{b_A p^{v_A}}{1+b_A p^{v_A}} + q_{B,sat}\frac{b_B p^{v_B}}{1+b_B p^{v_B}}$$

(1)

with T-dependent parameters b_A, and b_B

$$b_A = b_{A0}\exp\left(\frac{E_A}{RT}\right); \quad b_B = b_{B0}\exp\left(\frac{E_B}{RT}\right) \qquad (2)$$

The parameters are provided in Table S1.

4. Virial Graph Analysis.

Estimation of the isosteric heats of gas adsorption (Q_{st})

A virial-type expression comprising the temperature-independent parameters a_i and b_j was

employed to calculate the enthalpies of adsorption for C_3H_6 (at 296 K and 313 K) and C_2H_4 (at 273 K and 296 K) on ZJU-74a-Pd. In each case, the data were fitted using the equation:

$$\ln P = \ln N + 1/T\sum_{i=0}^{m} a_i N_i + \sum_{j=0}^{n} b_j N_j \quad (3)$$

Here, P is the pressure expressed in mmHg, N is the amount absorbed in mmol g^{-1}, T is the temperature in K, a_i and b_j are virial coefficients and m, n represent the number of coefficients required to adequately describe the isotherms (m and n were gradually increased till the contribution of extra added a and b coefficients was deemed to be statistically insignificant towards the overall fit. And the average value of the squared deviations from the experimental values was minimized). The values of the virial coefficients a_0 through a_m were then used to calculate the isosteric heat of absorption using the following expression:

$$Q_{st} = -R\sum_{i=0}^{m} a_i N_i \quad (4)$$

Q_{st} is the coverage-dependent isosteric heat of adsorption and R is the universal gas constant. The heat enthalpy of C_3H_6 and C_2H_4 sorption for ZJU-74a-Pd in this manuscript are determined by using the sorption data measured in the pressure range from 0-1 bar (at 273 K, 296 K, and 313 K).

5. IAST calculations.

The selectivity of preferential adsorption of component 1 (C_3H_6) over component 2 (C_2H_4) can be defined as

$$S_{ads} = \frac{q_1/q_2}{p_1/p_2} \quad (5)$$

In equation (5), q_1 and q_2 are the molar loadings in the adsorbed phase in equilibrium with the bulk gas phase with partial pressures p_1, and p_2. The component loadings and adsorption selectivity S_{ads} for 1/99 C_3H_6 (1)/C_2H_4 (2), 50/50 C_3H_6 (1)/C_2H_4 (2), and 10/90 C_3H_6 (1)/C_2H_4 (2) mixtures in ZJU-74a-Pd at 296 K were determined using IAST.

6. Computational results and details.

To obtain a reasonable binding site of gas molecules in ZJU-74a-Pd for subsequent modeling, Grand Canonical Monte Carlo (GCMC) simulations were performed in the MS modeling. The crystal structures of ZJU-74a-Pd were chosen for related simulations without further geometry optimization.

The framework and the individual C_3H_6 and C_2H_4 molecules were considered to be rigid during the simulation. Partial charges for atoms of guest-free ZJU-74a-Pd were derived from the QEq method and QEq_neutral1.0 parameter. The simulations were carried out at 298 K, adopting the locating task, the Metropolis method in the Sorption module, and the universal force field (UFF). The partial charges on the atoms of C_3H_6 (C1: −0.276e, C2: −0.141e, C3: −0.401e; H1: 0.142e, H2: 0.124e, H3: 0.146e, H4: 0.124e, H5: 0.141e, H6: 0.141e, where $e = 1.6022\times10^{-19}$ C is the elementary charge) and C_2H_4 (C: −0.293e; H:0.147e) were also derived from QEq method. The interaction energy between hydrocarbon molecules and framework was computed through the Coulomb and Lennard-Jones 6-12 (LJ) potentials. The cutoff radius was chosen as 12.5 Å for the LJ potential and the long-range electrostatic interactions were handled using the Ewald & Group summation method. The loading steps and the equilibration steps were 1×10^5, the production steps were 1×10^6. The binding energy between the frameworks and gas molecules was calculated using: $\Delta E = E_{(MOF)} + E_{(gas)} - E_{(MOF+gas)}$.

7. Gas equilibrium adsorption capacity.

The complete breakthrough of C_3H_6 was indicated by the downstream gas composition reaching that of the feed gas. Based on the mass balance, the gas adsorption capacities can be determined as follows:

$$q_i = \frac{C_i V}{22.4 \times m} \times \int_0^t \left(1 - \frac{F}{F_0}\right) dt \qquad (6)$$

Where q_i is the equilibrium adsorption capacity of gas i (mmol g^{-1}), C_i is the feed gas concentration, V is the volumetric feed flow rate (cm^3 min^{-1}), t is the adsorption time (min), F_0 and F are the inlet and outlet gas molar flow rates, respectively, and m is the mass of the adsorbent (g).

Notation

b_A	Langmuir-Freundlich constant for species *i* at adsorption site A, $\mathrm{Pa}^{-\nu_{iA}}$
b_B	Langmuir-Freundlich constant for species *i* at adsorption site B, $\mathrm{Pa}^{-\nu_{iB}}$
c_i	molar concentration of species *i* in a gas mixture, mol m^{-3}
c_{i0}	molar concentration of species *i* in the gas mixture at the inlet to adsorber, mol m^{-3}
E	energy parameter, J mol^{-1}
p_i	partial pressure of species *i* in the mixture, Pa
p_t	total system pressure, Pa
q_i	component molar loading of species *i*, mol kg^{-1}
Q_{st}	isosteric heat of adsorption, J mol^{-1}
T	absolute temperature, K

Greek letters

ν	Freundlich exponent, dimensionless
ρ	framework density, kg m^{-3}

Table S1. Dual-Langmuir-Freundlich parameter fits for C_3H_6 and C_2H_4 in ZJU-74a-Pd. The fits are based on experimental isotherm data at 296 K.

Gas	Site A			Site B		
	$q_{A,sat}$ mol kg^{-1}	b_{A0} kPa^{-vi}	v_A dimensionless	$q_{B,sat}$ mol kg^{-1}	b_{B0} kPa^{-vi}	v_B dimensionless
C_3H_6	1.54835	1.06598	2.09741	30.77694	0.02647	0.14881
C_2H_4	0.35181	2.31516E-14	12.33683	2.71704	0.16442	0.95504

Table S2. Comparison of physical parameters of C_3H_6 and C_2H_4.

	Kinetic diameter (Å)	Molecular dimensions (Å^3)[a]	Polarizability (10^{-25} cm^3)
C_3H_6	4.68	4.1 × 5.1 × 6.5	42.5
C_2H_4	4.16	3.2 × 4.2 × 4.8	62.6

[a] The dimensions of the gas molecules are reported in ref 3 and 4.

Table S3. Summary of adsorption capacities of C_3H_6 and C_2H_4 as well as C_2H_4/C_3H_6 (50/50, v/v) IAST selectivity and C_3H_6 adsorption heat for the indicated MOFs at RT under 1 bar.

Adsorbents	C_2H_4 uptake ($cm^3\ cm^{-3}$)	C_3H_6 uptake ($cm^3\ cm^{-3}$)			Selectivity (50/50)	Q_{st} (C_3H_6) ($kJ\ mol^{-1}$)	Temperature (K)	Ref.
	1 bar	0.01 bar	0.1 bar	1 bar				
**ZJU-74a-Pd**	**105.7**	**60.9**	**106.8**	**108.5**	**23.4**	**55.8**	**296**	**This work**
spe-MOF	34.4	-	22.4	166.8	7.7	29.6	298	5
MAC-4	89.2	35.2	96.8	136.5	9.5	25.3	298	6
Zn-BPZ-TATB	85.8	15.2	73.2	106	7.4	28.1	298	7
$Zn_2(oba)_2(dmimpym)$	53.5	16.3	59.1	83.8	15.6	33.3	298	8
iso-MOF-4	41.7	-	31.4	157	7.7	30.9	298	9
Mn-dtzip	88.4	23.6	77.4	249	8.6	35.1	298	10
$Cu_3(OH)_2(Me_2BPZ)_2$	47.7	-	36.7	126.5	7.4	30.3	298	11
Zn-BPZ-SA	63.9	7.8	46.6	68.3	4.8	33.6	298	12
MFM-202	41	-	38.9	101.5	8.4	33.0	293	13
UPC-33	29.1	-	19.4	88.1	5.7	48.9	298	14
CoV-bco-tpt	106.9	21.3	84.8	108	7.8	39.9	298	15
MIL-101(Cr)	42.8	10.9	31.6	131	6.6	34.3	298	16
Ni-MOF-74	161.1	53.5	133.8	187	3.3	54.5	298	17
NEM-7-Cu	30.7	-	58.2	80	8.6	36.9	298	18
LIFM-38	23.3	-	17.3	67	6.4	27.3	298	19
SIFSIX-1-Cu	-	22.3	91.8	133	-	-	296	20
UTSA-74(Zn)	-	5.4	22.5	154	-	-	298	20

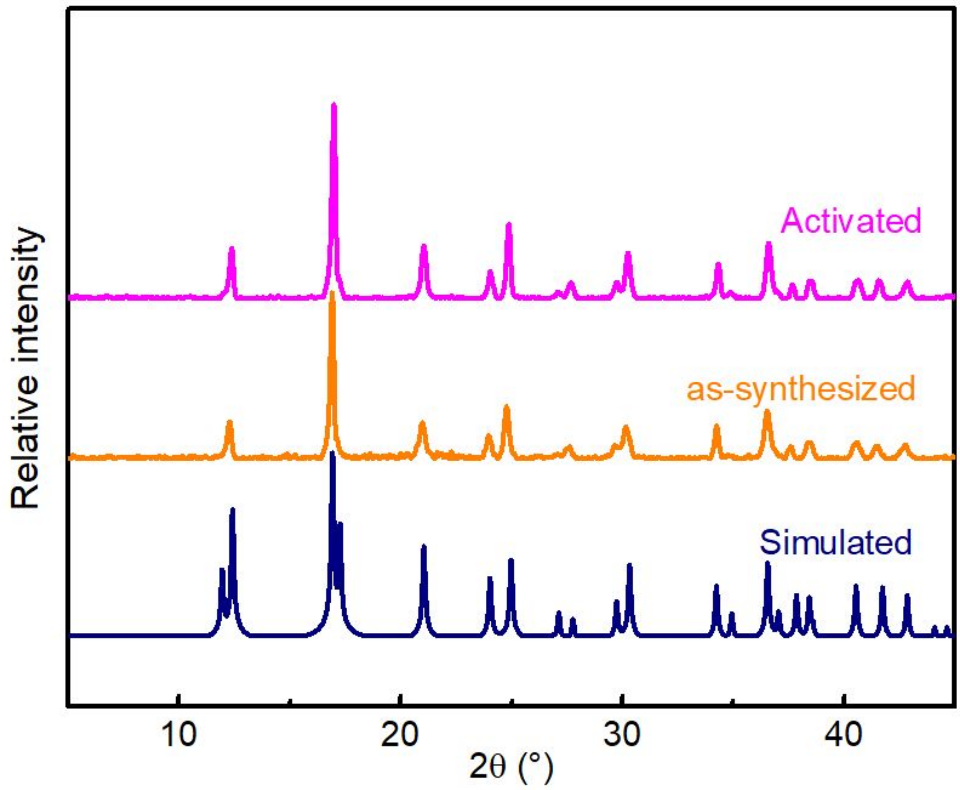

Figure S1. The simulated XRD pattern from the single-crystal X-ray structure of ZJU-74-Pd (navy), and the PXRD patterns of as-synthesized powder (orange) and activated ZJU-74-Pd (pink).

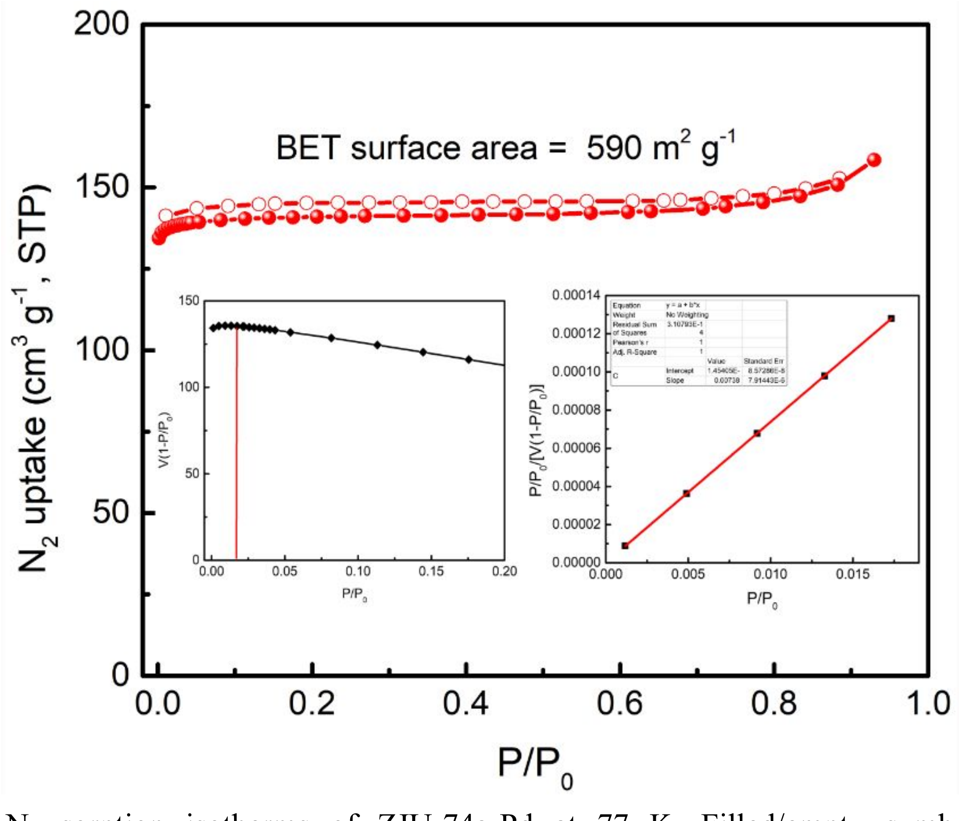

Figure S2. N_2 sorption isotherms of ZJU-74a-Pd at 77 K. Filled/empty symbols represent adsorption/desorption.

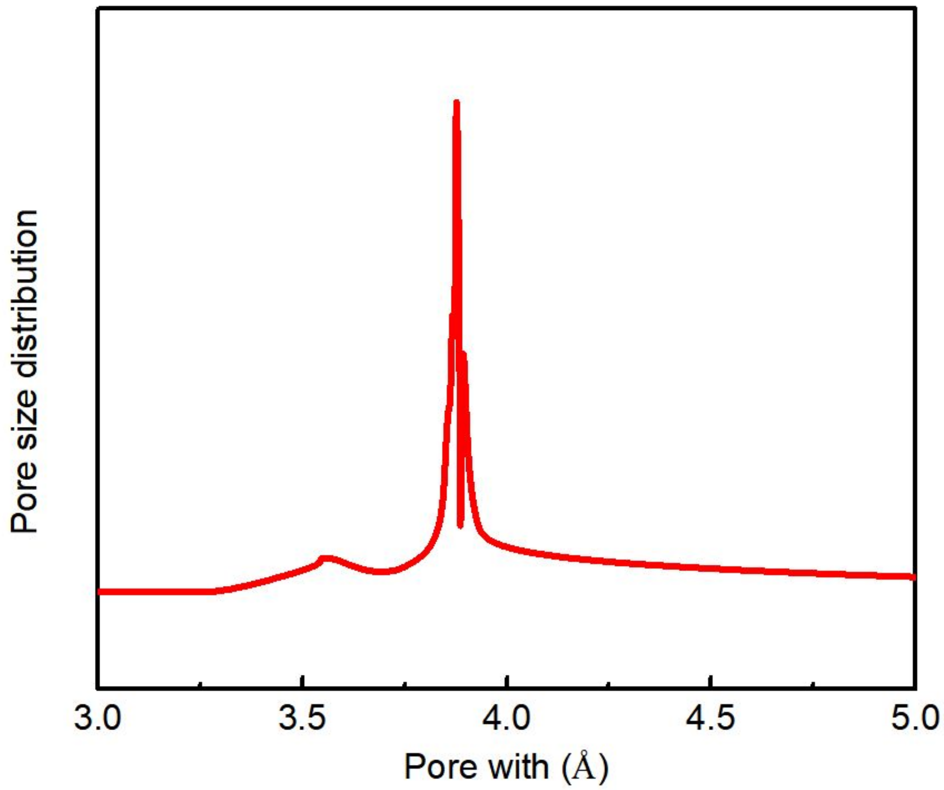

Figure S3. Pore size distribution of ZJU-74a-Pd determined by the Horvath−Kawazoe method based on low-dosing N_2 adsorption isotherms at 77 K.

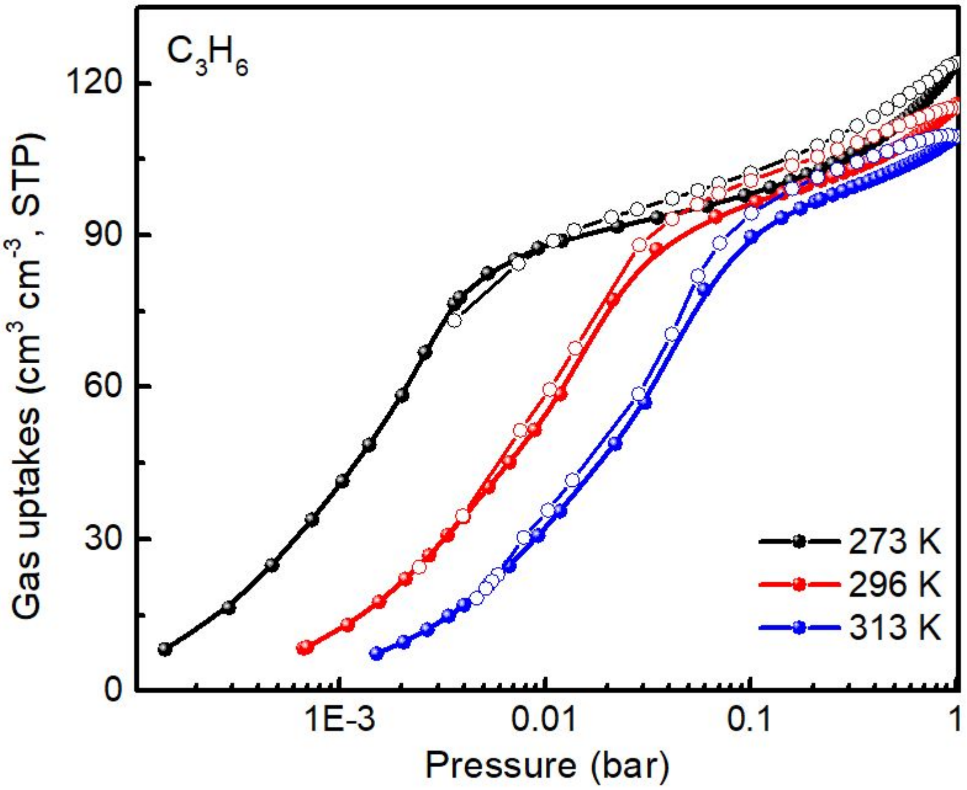

Figure S4. C_3H_6 adsorption (filled symbols) and desorption (empty symbols) isotherms of ZJU-74a-Pd at 273 K (black), 296 K (red), and 313 K (blue) up to 1 bar. The almost overlapped desorption isotherms at low pressure indicate that the adsorbed C_3H_6 molecules can be completely desorbed from ZJU-74a-Pd under vacuum conditions.

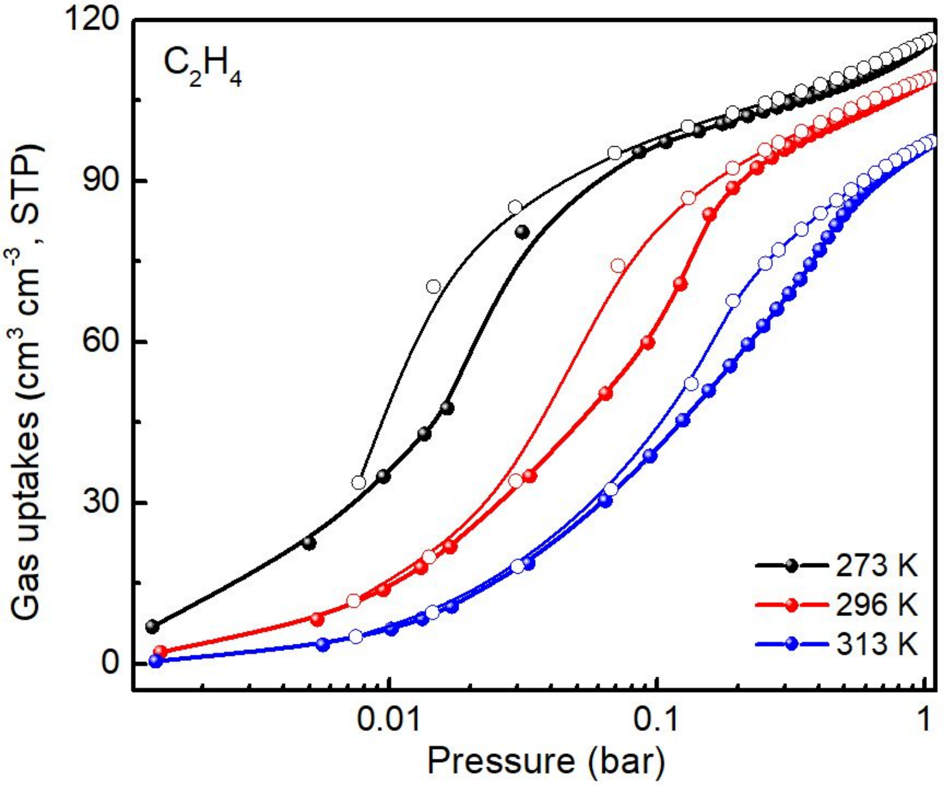

Figure S5. C_2H_4 adsorption (filled symbols) and desorption (empty symbols) isotherms of ZJU-74a-Pd at 273 K (black), 296 K (red), and 313 K (blue) up to 1 bar. The almost overlapped desorption isotherms at low pressure indicate that the adsorbed C_2H_4 molecules can be completely desorbed from ZJU-74a-Pd under vacuum conditions.

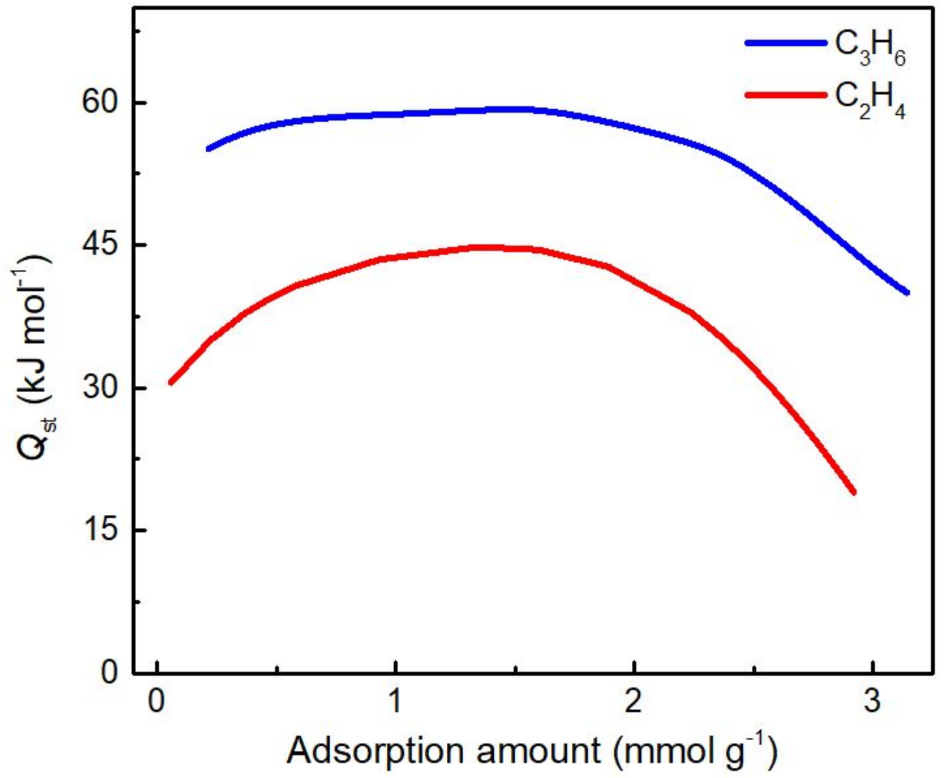

Figure S6. Adsorption heats (Q_{st}) of ZJU-74a-Pd for C_2H_4 (red) and C_3H_6 (blue).

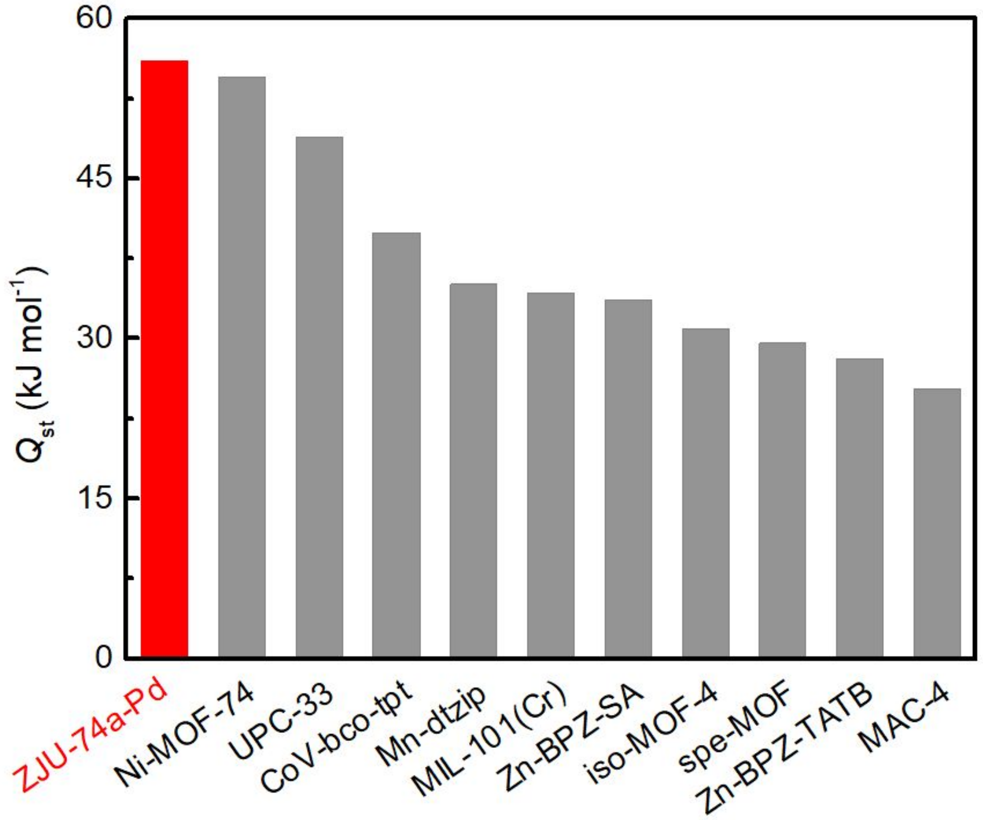

Figure S7. Comparison of initial C_3H_6 adsorption heats for ZJU-74a-Pd and other top-performing MOFs.

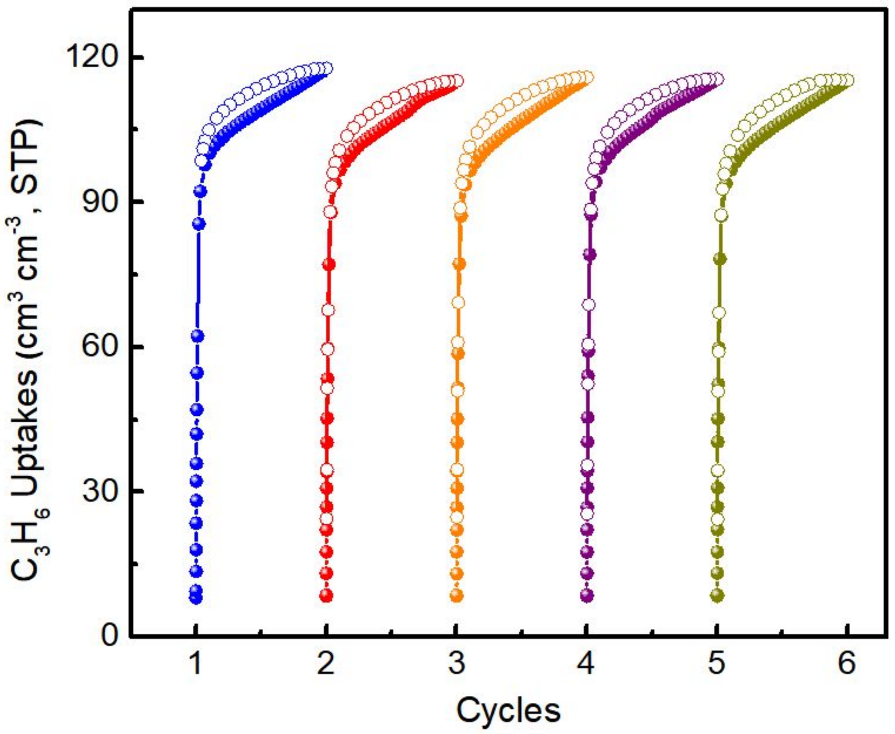

Figure S8. Cycling sorption measurements of C_3H_6 for ZJU-74a-Pd at 296 K up to 1 bar, indicating its good regeneration ability for C_3H_6 adsorption. Filled/empty symbols represent adsorption/desorption.

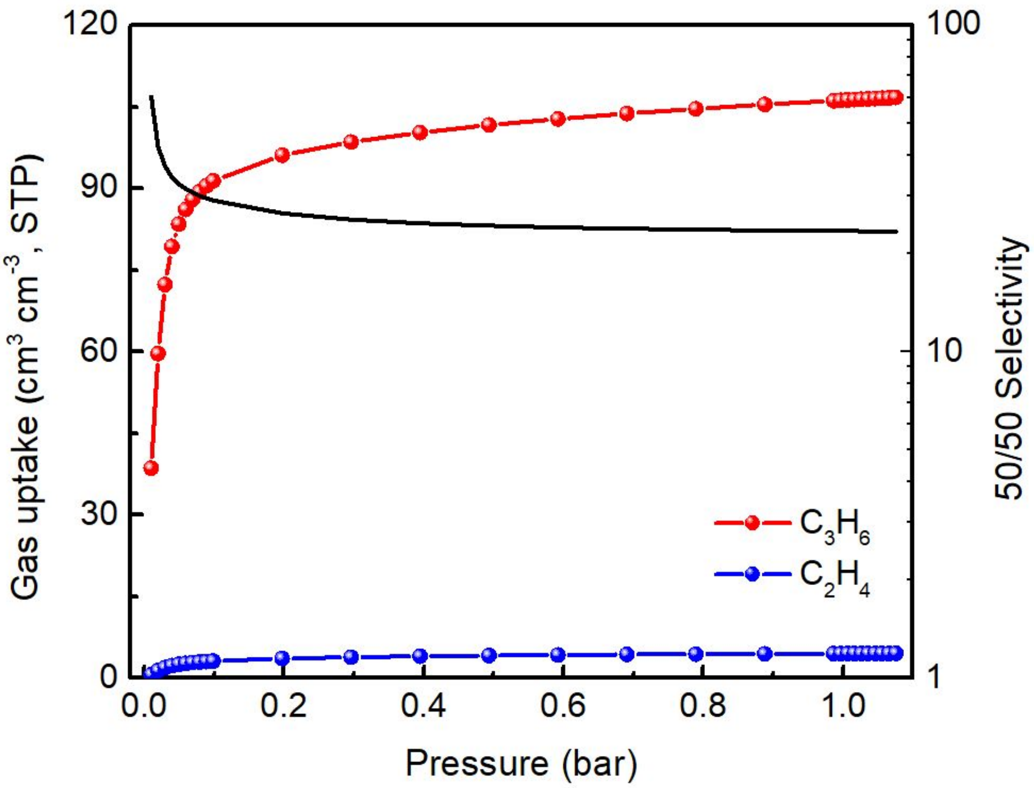

Figure S9. Mixture adsorption isotherms and selectivity of ZJU-74a-Pd predicted by the IAST method for a 50/50 (v/v) C_3H_6/C_2H_4 mixture at 296 K.

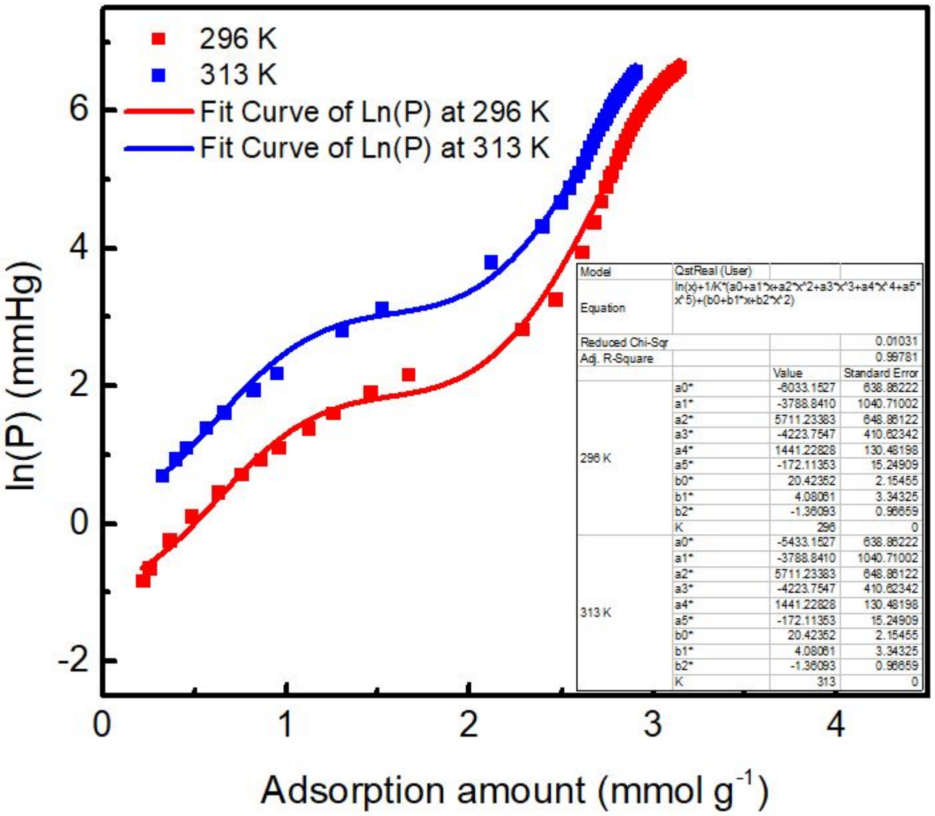

Figure S10. Virial fitting of the C_3H_6 adsorption isotherms for ZJU-74a-Pd.

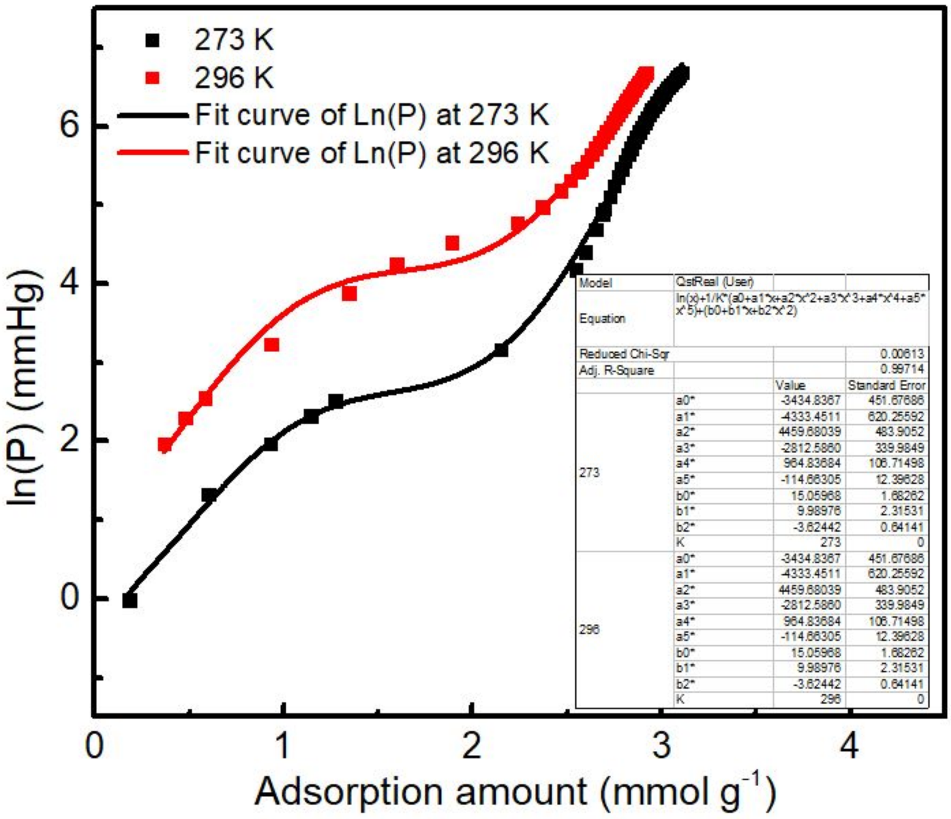

Figure S11. Virial fitting of the C_2H_4 adsorption isotherms for ZJU-74a-Pd.

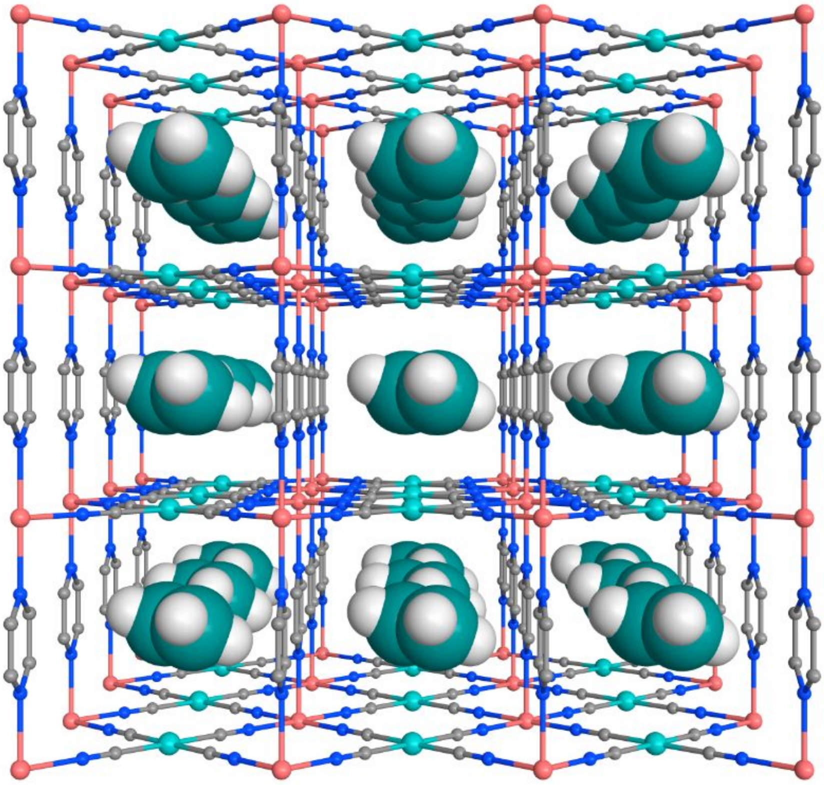

Figure S12. Illustration of C_2H_4 adsorption sites in ZJU-74a-Pd calculated by GCMC simulations.

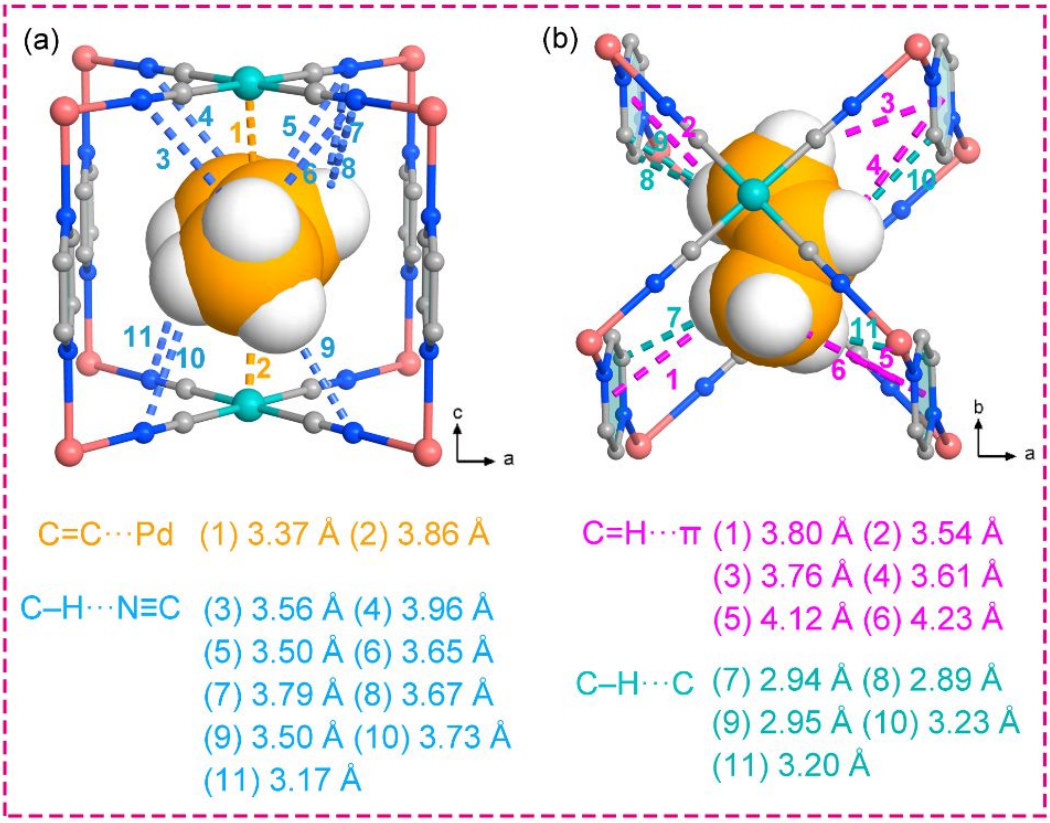

Figure S13. (a) Illustration of the π-complexation and multiple van der Waals interactions between the adsorbed C_3H_6 molecule and the single-molecule nano-trap viewed along *b* axis, and (b) viewed along *c* axis.

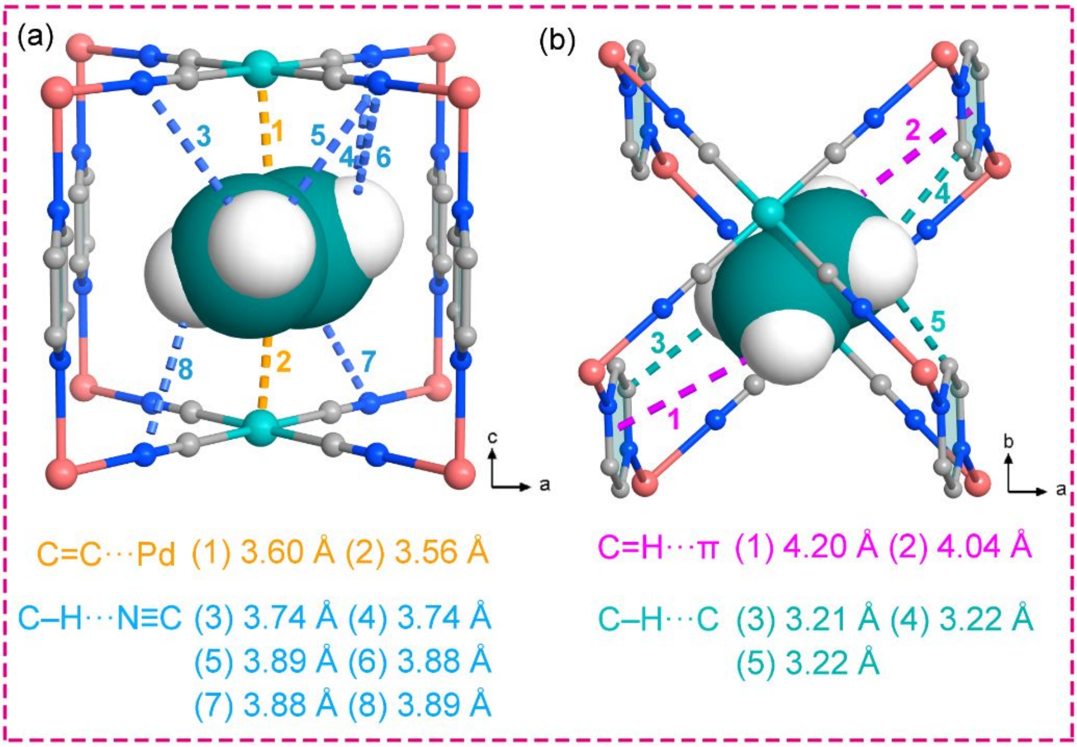

Figure S14. (a) Illustration of the π-complexation and multiple van der Waals interactions between the adsorbed C_2H_4 molecule and the framework viewed along *b* axis, and (b) viewed along *c* axis.

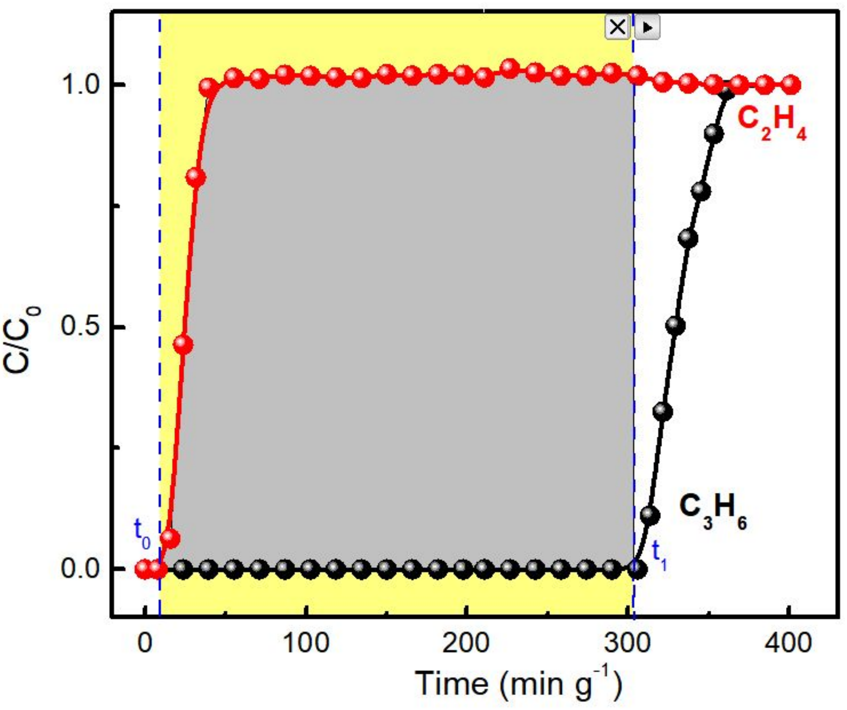

Figure S15. Breakthrough curves for a 1/99 C_3H_6/C_2H_4 mixture in ZJU-74a-Pd. The C_2H_4 productivity (17.4 mmol g^{-1}) is defined by the breakthrough amount of C_2H_4, which is calculated by integration of the breakthrough curves *f*(t) during a period from t_0 (7.8 min g^{-1}) to t_1 (306.7 min g^{-1}) (gray area).

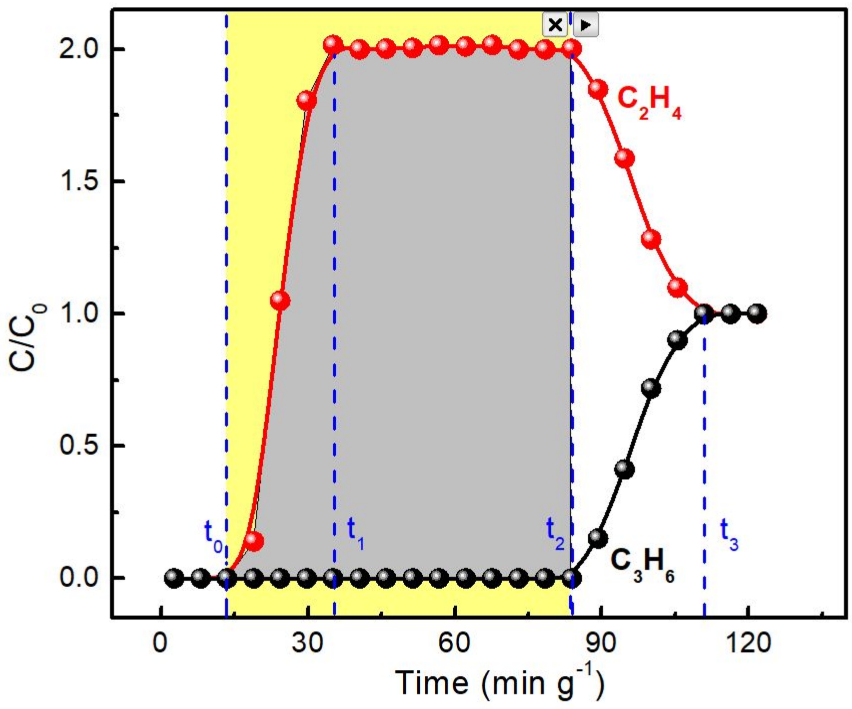

Figure S16. Breakthrough curves for a 50/50 C_3H_6/C_2H_4 mixture in ZJU-74a-Pd, during which C_2H_4 gas adsorbed from 0 to t_1 (35.2 min g^{-1}) and C_3H_6 gas adsorbed from 0 to t_3 (110.5 min g^{-1}). The dynamic C_3H_6/C_2H_4 selectivity is calculated to be 8.7. The C_2H_4 productivity (3.4 mmol g^{-1}) is defined by the breakthrough amount of C_2H_4, which is calculated by integration of the breakthrough curves *f*(t) during a period from t_0 (13.6 min g^{-1}) to t_2 (84.5 min g^{-1}) (gray area).

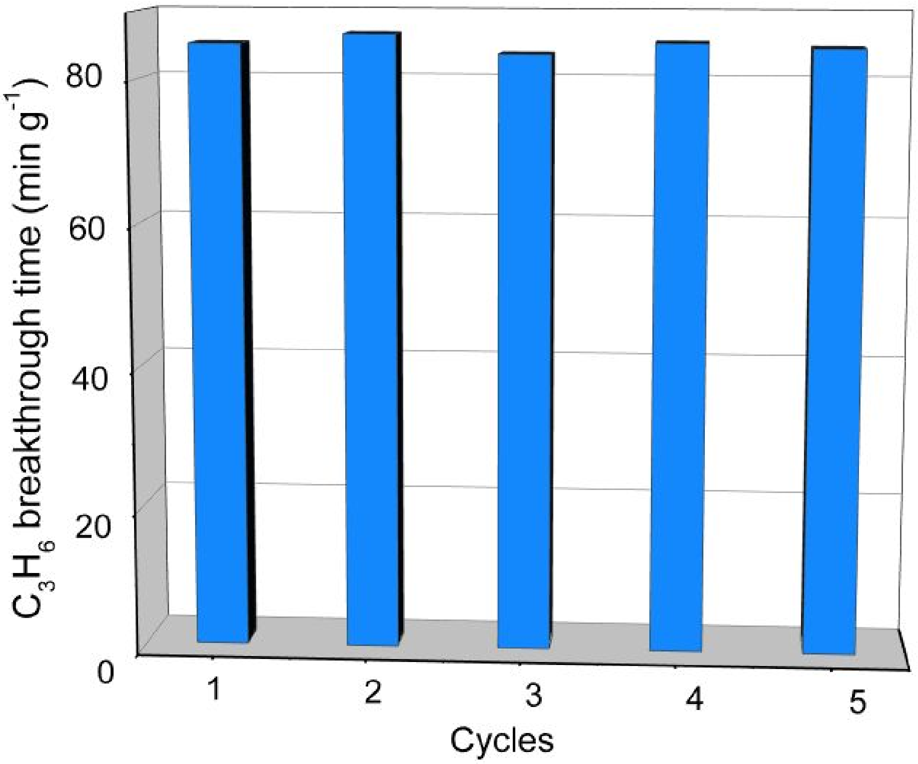

Figure S17. Cyclic breakthrough experiments on ZJU-74a-Pd for a C_3H_6/C_2H_4 (50/50) mixture, indicating that ZJU-74a-Pd maintained the C_3H_6 breakthrough time during the separation processes for at least five times.

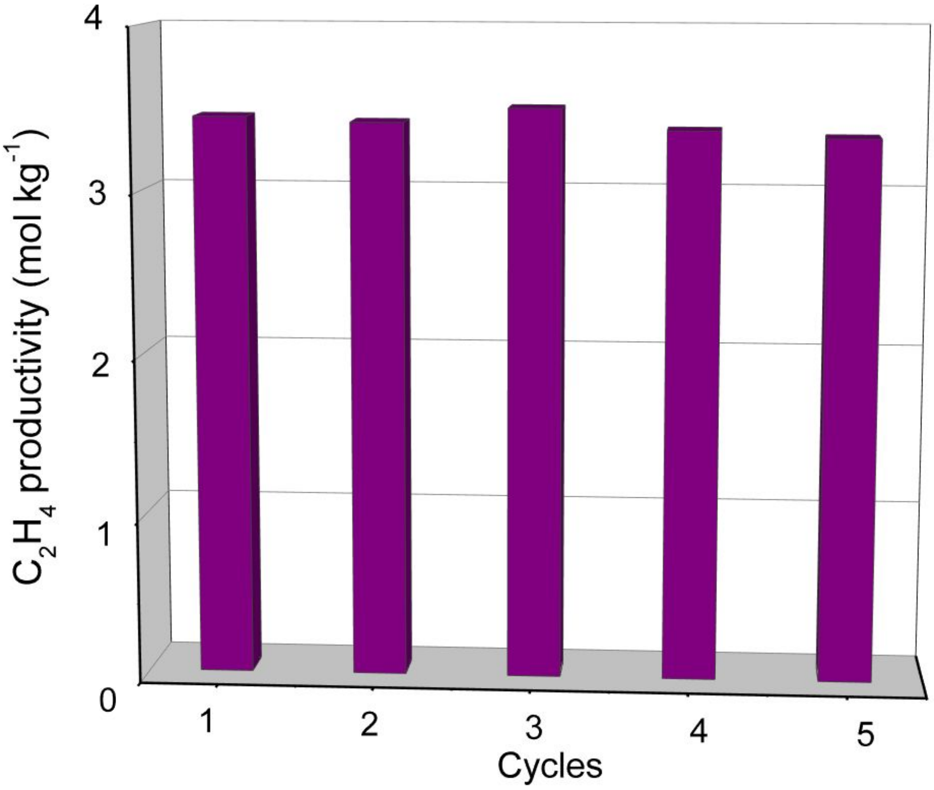

Figure S18. Cyclic breakthrough experiments on ZJU-74a-Pd for a C_3H_6/C_2H_4 (50/50) mixture, indicating that ZJU-74a-Pd maintained the C_2H_4 eluted amount during the separation processes for at least 5 times.

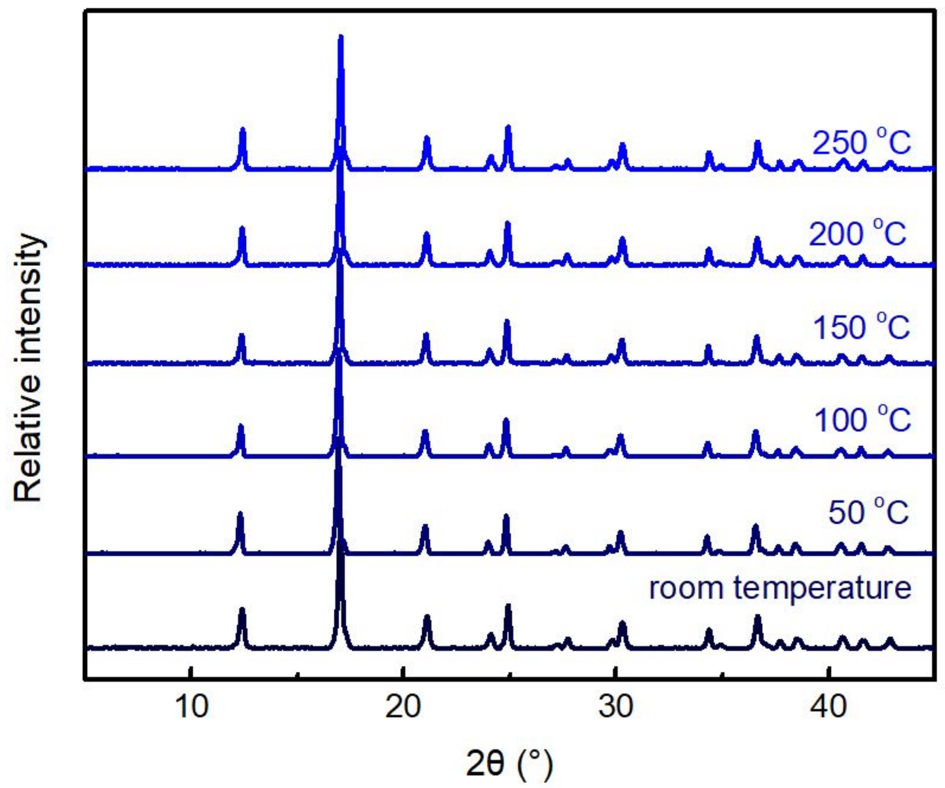

Figure S19. Variable-temperature PXRD patterns for ZJU-74-Pd, indicating its good thermal stability up to 250 °C.

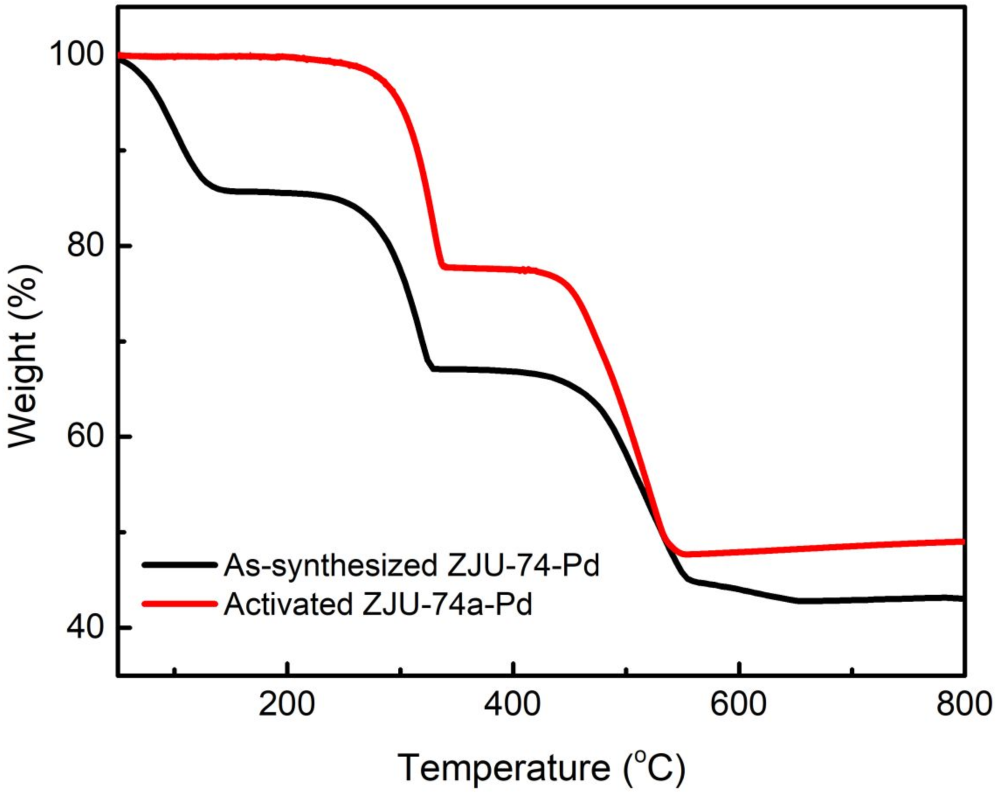

Figure S20. TGA curves of as-synthesized ZJU-74-Pd (black) and the activated ZJU-74a-Pd (red) under a flow of nitrogen at a rate of 5 °C min^{-1}.

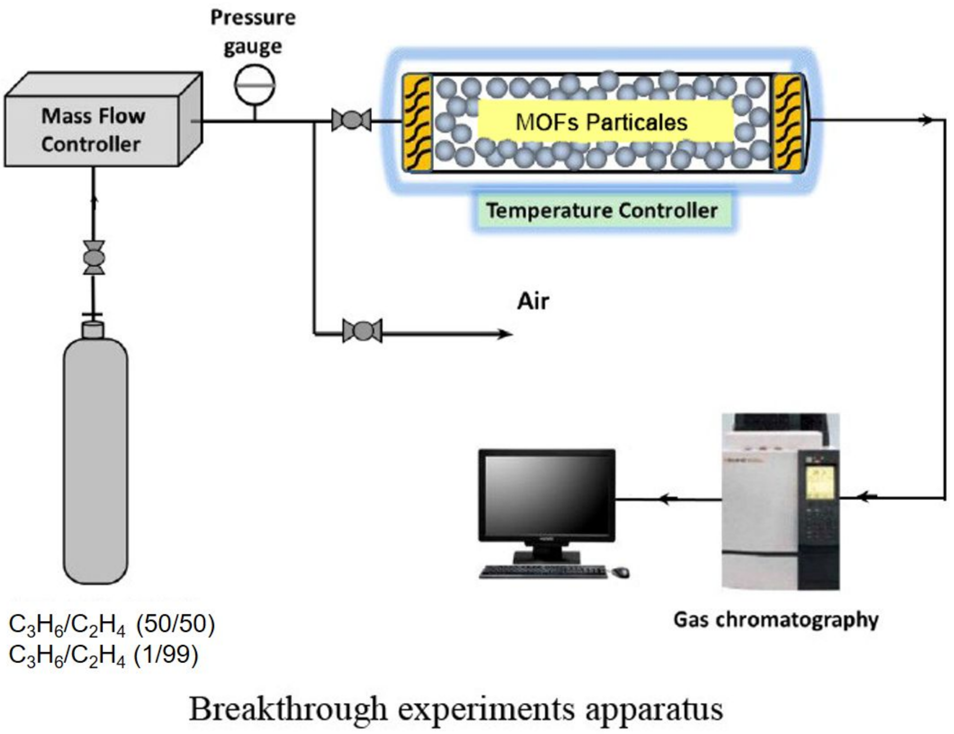

Figure S21. Schematic illustration of the apparatus for the breakthrough experiments.

References:

[1] Sheldrick, G. M. SHELXL-97, Program for the Refinement of Crystal Structures, University of Göttingen, Göttingen, Germany 1997.

[2] Pei, J.; Gu, X.-W.; Liang, C.-C.; Chen, B.; Li, B.; Qian, G. Robust and Radiation-Resistant Hofmann-Type Metal−Organic Frameworks for Record Xenon/Krypton Separation. *J. Am. Chem. Soc.* **2022**, *144*, 3200–3209.

[3] Cui, J.; Zhang, Z.; Yang, L.; Hu, J.; Jin, A.; Yang, Z.; Zhao, Y.; Meng, B.; Zhou, Y.; Wang, J.; Su, Y.; Wang, J.; Cui, X.; Xing, H. A Molecular Sieve with Ultrafast Adsorption Kinetics for Propylene Separation. *Science* **2024**, *383*, 179–183.

[4] Lin, R.-B.; Li, L.; Zhou, H.-L.; Wu, H.; He, C.; Li, S.; Krishna, R.; Li, J.; Zhou, W.; Chen, B. Molecular Sieving of Ethylene from Ethane Using a Rigid Metal–Organic Framework. *Nat. Mater*. **2018**, *17*, 1128–1133.

[5] Fang, H.; Zheng, B.; Zhang, Z.-H.; Li, H.-X.; Xue, D.-X.; Bai, J. Ligand-Conformer-Induced Formation of Zirconium–Organic Framework for Methane Storage and MTO Product Separation. *Angew. Chem. Int. Ed.* **2021**, *60*, 16521–16528.

[6] Wang, G.-D.; Li, Y.-Z.; Krishna, R.; Zhang, W.-Y.; Hou, L.; Wang, Y.-Y.; Zhu, Z. Scalable Synthesis of Robust MOF for Challenging Ethylene Purification and Propylene Recovery with Record Productivity. *Angew. Chem. Int. Ed.* **2024**, e202319978.

[7] Wang, G.-D.; Li, Y.-Z.; Shi, W.-J.; Hou, L.; Wang, Y.-Y.; Zhu, Z. Active Sites Decorated Nonpolar Pore-Based MOF for One-Step Acquisition of C_2H_4 and Recovery of C_3H_6. *Angew. Chem. Int. Ed.* **2023**, *62*, e202311654.

[8] Li, Y.-Z.; Wang, G.-D.; Krishna, R.; Yin, Q.; Zhao, D.; Qi, J.; Sui, Y.; Hou, L. A Separation MOF with O/N Active Sites in Nonpolar Pore for One-Step C_2H_4 Purification from C_2H_6 or C_3H_6 Mixtures. *Chem. Eng. J.* **2023**, *466*, 143056.

[9] Fan, W.; Wang, X.; Zhang, X.; Liu, X.; Wang, Y.; Kang, Z.; Dai, F.; Xu, B.; Wang, R.; Sun, D. Fine-Tuning the Pore Environment of the Microporous Cu-MOF for High Propylene Storage and Efficient Separation of Light Hydrocarbons. *ACS Cent. Sci.* **2019**, *5*, 1261–1268.

[10] Zhang, L.; Ma, L.-N.; Wang, G.-D.; Hou, L.; Zhu, Z.; Wang, Y.-Y. A New Honeycomb MOF for C_2H_4 Purification and C_3H_6 Enrichment by Separating Methanol to Olefin Products. *J. Mater. Chem. A* **2023**, *11*, 2343–2348.

[11] Zhen, G.; Liu, Y.; Zhou, Y.; Ji, Z.; Li, H.; Zou, S.; Zhang, W.; Li, Y.; Liu, Y.; Chen, C.; Wu, M. Water-Stable Microporous Bipyrazole-Based Framework for Efficient Separation of MTO Products. *ACS Appl. Mater. Interfaces* **2024**, *16*, 1179–1186.

[12] Wang, G.-D.; Krishna, R.; Li, Y.-Z.; Ma, Y.-Y.; Hou, L.; Wang, Y.-Y.; Zhu, Z. Rational Construction of Ultrahigh Thermal Stable MOF for Efficient Separation of MTO Products and Natural Gas. *ACS Materials Lett.* **2023**, *5*, 1091–1099.

[13] Gao, S.; Morris, C. G.; Lu, Z.; Yan, Y.; Godfrey, H. G. W.; Murray, C.; Tang, C. C.; Thomas, K. M.; Yang, S.; Schröder, M. Selective Hysteretic Sorption of Light Hydrocarbons in a Flexible Metal–Organic Framework Material. *Chem. Mater.* **2016**, *28*, 2331–2340.

[14] Fan, W.; Wang, Y.; Zhang, Q.; Kirchon, A.; Xiao, Z.; Zhang, L.; Dai, F.; Wang, R.; Sun, D. An Amino-Functionalized Metal–Organic Framework, Based on a Rare $Ba_{12}(COO)_{18}(NO_3)_2$ Cluster, for Efficient $C_3/C_2/C_1$ Separation and Preferential Catalytic Performance. *Chem. Eur. J.* **2018**, *24*, 2137–2143.

[15] Xiao, Y.; Hong, A. N.; Chen, Y.; Yang, H.; Wang, Y.; Bu, X.; Feng, P. Developing Water-Stable Pore-Partitioned Metal–Organic Frameworks with Multi-Level Symmetry for High-Performance Sorption Applications. *Small* **2023**, *19*, 2205119.

[16] Liu, X.-M.; Xie, L.-H.; Wu, Y. Efficient Propylene/Ethylene Separation in Highly Porous Metal–Organic Frameworks. *Materials* **2023**, *16*, 154.

[17] Wu, X.; Bao, Z.; Yuan, B.; Wang, J.; Sun, Y.; Luo, H.; Deng, S. Microwave Synthesis and Characterization of MOF-74 (M = Ni, Mg) for Gas Separation. *Microporous Mesoporous Mater.* **2013**, *180*, 114−122.

[18] Liu, X.; Hao, C.; Li, J.; Wang, Y.; Hou, Y.; Li, X.; Zhao, L.; Zhua, H.; Guo, W. An Anionic Metal–Organic Framework: Metathesis of Zinc(II) with Copper(II) for Efficient C_3/C_2 Hydrocarbon and Organic Dye Separation. *Inorg. Chem. Front.* **2018**, *5*, 2898–2905.

[19] Chen, C.-X.; Wei, Z.-W.; Qiu, Q.-F.; Fan, Y.-Z.; Cao, C.-C.; Wang, H.-P.; Jiang, J.-J.; Fenske, D.; Su, C.-Y. A Porous Zn(II)-Metal–Organic Framework Constructed from Fluorinated Ligands for Gas Adsorption. *Cryst. Growth Des.* **2017**, *17*, 1476–1479.

[20] Li, L.; Wen, H.-M.; He, C.; Lin, R.-B.; Krishna, R.; Wu, H.; Zhou, W.; Li, J.; Li, B.; Chen, B. A Metal–Organic Framework with Suitable Pore Size and Specific Functional Sites for the Removal of Trace Propyne from Propylene. *Angew. Chem. Int. Ed.* **2018**, *57*,15183–15188.
